# Non-pharmaceutical interventions to reduce COVID-19 transmission in the UK: a rapid mapping review and interactive evidence gap map

**DOI:** 10.1093/pubmed/fdae025

**Published:** 2024-02-29

**Authors:** D Duval, B Evans, A Sanders, J Hill, A Simbo, T Kavoi, I Lyell, Z Simmons, M Qureshi, N Pearce-Smith, C R Arevalo, C R Beck, R Bindra, I Oliver

**Affiliations:** Research, Evidence and Knowledge Division, UK Health Security Agency (UKHSA), London E14 5EA, UK; Research, Evidence and Knowledge Division, UK Health Security Agency (UKHSA), London E14 5EA, UK; Research, Evidence and Knowledge Division, UK Health Security Agency (UKHSA), London E14 5EA, UK; Clinical and Public Health Response Division, UKHSA, London E14 5EA, UK; Evaluation and Epidemiological Science Division, UKHSA, Colindale NW9 5EQ, UK; Cheshire and Merseyside Health Protection Team, UKHSA, Liverpool L3 1DS, UK; Greater Manchester Health Protection Team, UKHSA, Manchester M1 3BN, UK; Research, Evidence and Knowledge Division, UK Health Security Agency (UKHSA), London E14 5EA, UK; Clinical and Public Health Response Division, UKHSA, London E14 5EA, UK; Research, Evidence and Knowledge Division, UK Health Security Agency (UKHSA), London E14 5EA, UK; Research, Evidence and Knowledge Division, UK Health Security Agency (UKHSA), London E14 5EA, UK; Evaluation and Epidemiological Science Division, UKHSA, Salisbury SP4 0JG, UK; Clinical and Public Health Response Division, UKHSA, London E14 5EA, UK; Director General Science and Research and Chief Scientific Officer, UKHSA, London E14 5EA, UK

**Keywords:** COVID-19, non-pharmaceutical interventions

## Abstract

**Background:**

Non-pharmaceutical interventions (NPIs) were crucial in the response to the COVID-19 pandemic, although uncertainties about their effectiveness remain. This work aimed to better understand the evidence generated during the pandemic on the effectiveness of NPIs implemented in the UK.

**Methods:**

We conducted a rapid mapping review (search date: 1 March 2023) to identify primary studies reporting on the effectiveness of NPIs to reduce COVID-19 transmission. Included studies were displayed in an interactive evidence gap map.

**Results:**

After removal of duplicates, 11 752 records were screened. Of these, 151 were included, including 100 modelling studies but only 2 randomized controlled trials and 10 longitudinal observational studies.

Most studies reported on NPIs to identify and isolate those who are or may become infectious, and on NPIs to reduce the number of contacts. There was an evidence gap for hand and respiratory hygiene, ventilation and cleaning.

**Conclusions:**

Our findings show that despite the large number of studies published, there is still a lack of robust evaluations of the NPIs implemented in the UK. There is a need to build evaluation into the design and implementation of public health interventions and policies from the start of any future pandemic or other public health emergency.

## Introduction

Non-pharmaceutical interventions (NPIs), also known as public health and social measures, are interventions used to reduce the spread of infectious pathogens that are not based on medicinal products such as drugs or vaccines.^1^ The term NPI encompasses a wide variety of measures such as physical distancing, face covering use, asymptomatic testing and setting closures.

NPIs played a particularly important role in the early stages of the COVID-19 pandemic, before effective treatment and vaccination became available, although their role remained important throughout.^2^^,^^3^ In contrast to pharmaceutical interventions, there is a lack of strong evidence on the effectiveness of NPIs to reduce COVID-19 transmission, and for many NPIs the scientific consensus shifted over the course of the pandemic.^4^

Whilst this can be partly explained by the evolving epidemiology of COVID-19 throughout the pandemic, including changes in seroprevalence and variants, there are specific limitations to this evidence-base.^4^^,^^5^ One of the limitations is that the evidence for effectiveness of NPIs is mainly based on observational studies, which are at higher risk of bias than randomized controlled trials (RCTs). However, undertaking RCTs in the pandemic context can be challenging and may not be feasible or ethical.

Modelling studies were also used to assess effectiveness of NPIs, especially in the early stage of the pandemic. However, modelling studies may be based on assumptions and hypothetical scenarios, and do not usually take into account the evolving epidemiology and human behaviours.

Despite the volume of research published,^1^^,^^6^^–^^13^ interpreting the evidence on effectiveness of NPIs remains challenging due to differences in methodologies,^14^ in implementation across countries, and in socio-demographics and healthcare systems.^15^ In addition, NPIs were implemented in ‘packages’ (combination of different NPIs), which varied in terms of NPIs included, and how and when they were implemented across countries. NPIs have complex combined effects, which makes the interpretation of the evidence and the assessment of the effectiveness of individual NPIs even more challenging.^1^^,^^3^^,^^9^

Behavioural changes are also an important consideration, and it is not always possible to distinguish the impact of NPI policies implemented by a government from wider behavioural changes in the pandemic context.^4^^,^^5^

To reduce some of the heterogeneity observed due to differences between countries, we focused here on evidence from the UK. Similarly, we did not consider evidence from health and social care settings due to differences in policy and implementation between settings (for instance, in the UK the use of surgical masks was only mandatory in healthcare settings), differences in transmission risk and risk of severe COVID-19 outcomes, and also in knowledge and experience of NPI use.

In this context, we conducted a rapid mapping review to identify and categorize primary studies evaluating the effectiveness of NPIs implemented in community settings to reduce the transmission of COVID-19 in the UK. This paper is a summary of the full report, available elsewhere.^16^

## Methods

We used streamlined systematic methods^17^ to conduct a rapid mapping review according to the PRISMA checklist for scoping reviews,^18^ which is also applicable to mapping reviews.^19^ We produced a protocol before the literature searching began. Modifications to the protocol are summarized in Supplementary Data S1.

### Eligibility criteria

We included primary studies reporting on the effectiveness of NPIs to reduce the transmission of COVID-19. Any NPI implemented in community (non-healthcare) settings in the UK was considered for inclusion, and NPI were grouped into five categories:

- Measures to reduce infection risk at individual level (face covering use, physical distancing, hand and respiratory hygiene, cleaning and ventilation)
- Measures to identify and isolate those who are or may become infectious (contact tracing, asymptomatic and symptomatic testing, isolation of cases and contacts, and test and release strategies)
- Measures to reduce the numbers of contacts, including limitation of social contacts and restrictions of large gatherings, cohorting measures in non-healthcare settings such as school bubbles or household support bubbles, setting closures (workplace, hospitality and schools), and lockdown and tiered restrictions as implemented in the UK
- Measures to protect the most vulnerable (shielding)
- Travel restrictions (such as travel corridors) and border measures (such as testing and isolation of travellers arriving in the UK)

Studies published in peer-reviewed journals or as preprints between 1 January 2020 and 28 February 2023 were considered for inclusion. Any outcomes related to the impact of the intervention on the COVID-19 pandemic were considered, including COVID-19 transmission, COVID-19 cases, as well as COVID-19 hospitalization and COVID-19 mortality when used as proxies for transmission. Behavioural outcomes such as adherence or attitudes, and impact on school or work attendance were also considered when directly linked to effectiveness of NPIs.

We excluded studies reporting on:

- NPIs specific to healthcare settings
- Effectiveness of ‘packages’ or ‘stringency’ of measures if they did not report on the effectiveness of individual NPIs
- The performance of specific tests or products in controlled environments (‘efficacy’) rather than reporting on real-world performance (‘effectiveness’)

See Supplementary Data S2 for inclusion and exclusion criteria.

### Search strategy

We identified studies using two main methods. For studies published in 2020, we screened relevant systematic reviews^7^^–^^9^^,^^12^^,^^20^^–^^40^ identified via a scoping exercise.^16^ For studies published from 1 January 2021 to 28 February 2023, we conducted searches using the databases Ovid Medline, Ovid Embase, NIH Covid Portfolio (for preprints from medRxiv, aRxiv and Research Square preprints) and CoronaCentral.^41^

Additional sources included bibliographies from previous reviews conducted by UK Health Security Agency (UKHSA),^42^ and the UKHSA research portal.^43^

The search strategies were drafted by an information scientist and peer reviewed by a second information scientist. A validated geographical search filter was used to limit the search results to UK studies.^44^^,^^45^ See Supplementary Data S3 for the search strategies.

### Screening

We used EPPI-Reviewer Web Version 4 (EPPI-R)^46^ for title and abstract screening of database search results (15% screened in duplicate and the remaining 85% screened by one reviewer). Primary studies identified by other methods were screened on title and abstract by one reviewer before being added to EPPI-R.

Full-text screening was done by one reviewer and verified by a second reviewer. Disagreements were resolved through discussion with a third reviewer.

### Data charting

For the mapping, we coded the study designs, NPIs and outcomes in EPPI-R (see Supplementary Data S4 for the list of codes).

We also extracted in Microsoft Word additional information for each study, including study objectives, settings, participants or population, time period and other relevant methodological information.

Data extraction and coding were undertaken by one reviewer and checked by a second. Discrepancies were resolved by discussion.

Critical appraisal was not performed as the main aim of the mapping review was to identify and categorize the evidence.^47^ Reviewers did not contact authors for information relating to studies.

### Synthesis of results

We provided a narrative summary of the evidence identified, including breakdown by study design and NPI.

We produced an interactive evidence gap map (EPPI-Mapper^48^) in which studies were plotted by NPI and study design, with the outcomes represented as mosaic tiles.^49^

## Results

### Sources of evidence

The database searches returned 15 846 records. After removal of duplicates, 11 752 records were screened on title and abstract. Of these, 607 full-text articles were assessed for eligibility and 138 were included.

Thirteen additional primary studies were identified: 11 by screening reference lists of systematic reviews and 2 through the additional sources screened.

**Fig. 1:**
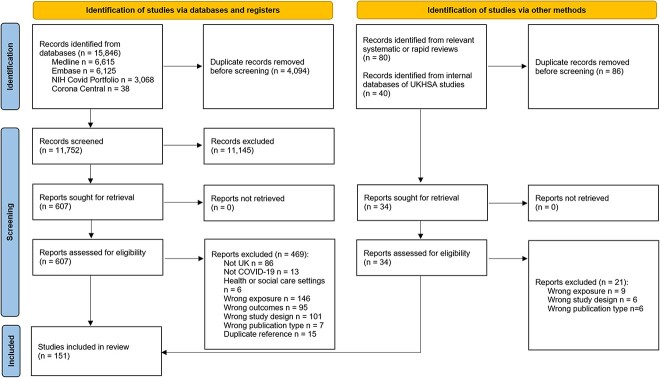
PRISMA diagram.

In total, 151 studies were included in this review (see Fig. 1 for PRISMA diagram and Supplementary Data S5 for list of excluded studies).

### Study characteristics

Characteristics of the studies identified are provided in Table 1 and data charting in Supplementary Data S6.

**Table 1:** Characteristics of evidence identified (*N* = 151)

	*n*	%	References
**Publication type**			
Peer-reviewed publication	132	87	^50^ ^–^ ^52^ ^,^ ^54^ ^,^ ^56^ ^–^ ^59^ ^,^ ^61^ ^–^ ^66^ ^,^ ^68^ ^–^ ^70^ ^,^ ^72^ ^–^ ^78^ ^,^ ^80^ ^–^ ^90^ ^,^ ^92^ ^–^ ^124^ ^,^ ^126^ ^,^ ^128^ ^–^ ^132^ ^,^ ^135^ ^–^ ^137^ ^,^ ^139^ ^–^ ^153^ ^,^ ^155^^,^^157^^,^^159^^–^^161^^,^^163^^–^^175^^,^^178^^–^^195^^,^^197^^–^^200^
Preprint	19	13	^53^ ^,^ ^55^ ^,^ ^60^ ^,^ ^67^ ^,^ ^71^ ^,^ ^79^ ^,^ ^91^ ^,^ ^125^ ^,^ ^127^ ^,^ ^133^ ^,^ ^134^ ^,^ ^138^ ^,^ ^154^ ^,^ ^156^ ^,^ ^158^ ^,^ ^162^ ^,^ ^176^ ^,^ ^177^ ^,^ ^196^
**Study type**			
Randomized controlled trials	2	1	^150^ ^,^ ^151^
Non-randomized controlled trials	0	0	—
Longitudinal studies	10	7	^152^ ^–^ ^161^
Cross-sectional studies	12	8	^162^ ^–^ ^173^
Ecological studies	12	8	^174^ ^–^ ^185^
Modelling studies	100	66	^50^ ^–^ ^149^
Mixed-methods studies	5	3	^186^ ^–^ ^190^
Qualitative studies	10	7	^191^ ^–^ ^200^
**NPI category**			
Measures to identify and isolate those who are infectious or may become infectious	80	53	^52^ ^,^ ^53^ ^,^ ^57^ ^,^ ^60^ ^,^ ^61^ ^,^ ^65^ ^,^ ^68^ ^,^ ^71^ ^–^ ^73^ ^,^ ^77^ ^–^ ^79^ ^,^ ^81^ ^,^ ^85^ ^–^ ^88^ ^,^ ^90^ ^,^ ^91^ ^,^ ^93^ ^,^ ^94^ ^,^ ^101^ ^,^ ^104^ ^–^ ^106^ ^,^ ^108^ ^–^ ^110^ ^,^ ^115^ ^,^ ^118^ ^,^ ^124^ ^–^ ^126^ ^,^ ^128^ ^–^ ^130^ ^,^ ^133^ ^,^ ^138^ ^–^ ^142^ ^,^ ^144^ ^,^ ^147^ ^,^ ^149^ ^–^ ^156^ ^,^ ^159^ ^,^ ^160^ ^,^ ^164^ ^,^ ^167^ ^–^ ^169^ ^,^ ^171^ ^–^ ^173^ ^,^ ^175^ ^,^ ^176^ ^,^ ^178^ ^,^ ^179^ ^,^ ^182^ ^,^ ^184^ ^,^ ^186^ ^,^ ^188^ ^,^ ^189^ ^,^ ^191^ ^,^ ^193^ ^–^ ^200^
Measures to reduce the numbers of contacts	71	47	^50^ ^,^ ^51^ ^,^ ^53^ ^–^ ^56^ ^,^ ^61^ ^–^ ^68^ ^,^ ^71^ ^,^ ^72^ ^,^ ^74^ ^–^ ^76^ ^,^ ^82^ ^,^ ^84^ ^,^ ^86^ ^,^ ^92^ ^,^ ^93^ ^,^ ^95^ ^–^ ^101^ ^,^ ^103^ ^,^ ^106^ ^–^ ^109^ ^,^ ^111^ ^,^ ^112^ ^,^ ^114^ ^,^ ^116^^,^^117^^,^^119^^,^^121^^–^^123^^,^^126^^,^^132^^,^^136^^,^^137^^,^^139^^–^^141^^,^^143^^,^^145^^–^^148^^,^^153^^,^^161^^,^^163^^,^^165^^,^^166^^,^ ^174^^,^^175^^,^^177^^,^^180^^,^^181^^,^^183^^,^^185^^,^^187^^,^^191^
Measures aimed at reducing infection risk at individual level	19	13	^65^ ^,^ ^67^ ^,^ ^68^ ^,^ ^72^ ^,^ ^76^ ^,^ ^80^ ^,^ ^83^ ^,^ ^89^ ^,^ ^102^ ^,^ ^108^ ^,^ ^113^ ^,^ ^115^ ^,^ ^119^ ^,^ ^120^ ^,^ ^140^ ^,^ ^143^ ^,^ ^163^ ^,^ ^166^ ^,^ ^170^
Travel and border restrictions	12	8	^58^ ^,^ ^59^ ^,^ ^67^ ^,^ ^69^ ^,^ ^70^ ^,^ ^75^ ^,^ ^127^ ^,^ ^134^ ^,^ ^162^ ^,^ ^175^ ^,^ ^190^ ^,^ ^192^
Measures to protect the most vulnerable	9	6	^61^ ^,^ ^72^ ^,^ ^86^ ^,^ ^126^ ^,^ ^131^ ^,^ ^135^ ^,^ ^153^ ^,^ ^157^ ^,^ ^158^
Studies reporting on more than one NPI	44	29	^52^ ^,^ ^53^ ^,^ ^61^ ^,^ ^65^ ^,^ ^67^ ^,^ ^68^ ^,^ ^71^ ^,^ ^72^ ^,^ ^75^ ^,^ ^76^ ^,^ ^83^ ^,^ ^86^ ^,^ ^87^ ^,^ ^90^ ^,^ ^93^ ^,^ ^94^ ^,^ ^101^ ^,^ ^104^ ^,^ ^106^ ^,^ ^108^ ^,^ ^109^ ^,^ ^111^ ^,^ ^113^ ^,^ ^115^^,^^118^^,^^119^^,^^126^^,^^128^^,^^129^^,^^132^^,^^139^^–^^141^^,^^143^^,^^144^^,^^146^^,^^147^^,^^153^^,^^161^^,^^163^^,^^166^^,^^171^^,^^175^^,^^191^
**Outcome**			
COVID-19 transmission	74	49	^50^ ^–^ ^53^ ^,^ ^57^ ^,^ ^60^ ^–^ ^63^ ^,^ ^65^ ^,^ ^68^ ^–^ ^70^ ^,^ ^72^ ^–^ ^74^ ^,^ ^78^ ^,^ ^79^ ^,^ ^81^ ^–^ ^84^ ^,^ ^86^ ^–^ ^88^ ^,^ ^90^ ^,^ ^93^ ^,^ ^95^ ^,^ ^97^ ^,^ ^98^ ^,^ ^100^ ^–^ ^107^ ^,^ ^109^ ^,^ ^110^^,^^112^^,^^113^^,^^116^^–^^118^^,^^121^^–^^125^^,^^127^^,^^128^^,^^130^^,^^132^^,^^133^^,^^135^^,^^138^^–^^142^^,^^144^^,^^146^^,^^147^^,^ ^149^^–^^151^^,^^156^^,^^159^^,^^162^^,^^165^^,^^175^^,^^177^^,^^178^
COVID-19 cases	59	39	^50^ ^,^ ^53^ ^–^ ^55^ ^,^ ^58^ ^,^ ^59^ ^,^ ^64^ ^,^ ^67^ ^,^ ^68^ ^,^ ^71^ ^–^ ^73^ ^,^ ^75^ ^–^ ^78^ ^,^ ^82^ ^,^ ^86^ ^,^ ^89^ ^,^ ^91^ ^,^ ^92^ ^,^ ^94^ ^,^ ^96^ ^,^ ^99^ ^,^ ^100^ ^,^ ^108^ ^,^ ^115^ ^,^ ^119^^–^^122^^,^^126^^,^^129^^,^^131^^,^^132^^,^^134^^,^^137^^,^^138^^,^^143^^,^^145^^,^^148^^,^^152^^–^^155^^,^^157^^,^^158^^,^^161^^,^^163^^,^^166^^,^ ^167^^,^^174^^,^^176^^–^^180^^,^^182^^,^^183^
COVID-19-related mortality	35	23	^50^ ^,^ ^54^ ^,^ ^56^ ^,^ ^66^ ^,^ ^68^ ^,^ ^71^ ^,^ ^72^ ^,^ ^75^ ^,^ ^77^ ^,^ ^86^ ^,^ ^89^ ^,^ ^96^ ^,^ ^99^ ^,^ ^100^ ^,^ ^107^ ^,^ ^113^ ^,^ ^114^ ^,^ ^121^ ^,^ ^122^ ^,^ ^126^ ^,^ ^131^ ^,^ ^132^ ^,^ ^136^ ^,^ ^145^^,^^146^^,^^148^^,^^155^^–^^157^^,^^174^^,^^176^^,^^179^^–^^182^
COVID-19 hospitalization	24	16	^50^ ^,^ ^61^ ^,^ ^66^ ^,^ ^72^ ^,^ ^77^ ^,^ ^80^ ^,^ ^85^ ^,^ ^89^ ^,^ ^98^ ^–^ ^100^ ^,^ ^114^ ^,^ ^126^ ^,^ ^131^ ^,^ ^132^ ^,^ ^137^ ^,^ ^146^ ^,^ ^148^ ^,^ ^156^ ^,^ ^157^ ^,^ ^174^ ^,^ ^176^ ^,^ ^179^^,^^184^
Behavioural outcomes	30	20	^81^ ^,^ ^150^ ^,^ ^159^ ^,^ ^160^ ^,^ ^164^ ^,^ ^165^ ^,^ ^167^ ^–^ ^173^ ^,^ ^179^ ^,^ ^185^ ^–^ ^200^
Lost time (school or work)	12	8	^60^ ^,^ ^79^ ^,^ ^101^ ^,^ ^104^ ^,^ ^106^ ^,^ ^115^ ^,^ ^125^ ^,^ ^128^ ^,^ ^141^ ^,^ ^150^ ^,^ ^151^ ^,^ ^160^

Of the 151 studies, 100 were modelling.^50^^–^^149^ Of the remaining studies, 2 were RCTs,^150^^,^^151^ 22 were individual-level observational (10 longitudinal^152^^–^^161^ and 12 cross-sectional^162^^–^^173^), 12 were ecological,^174^^–^^185^ 5 were mixed-methods^186^^–^^190^ and 10 were qualitative studies.^191^^–^^200^

In terms of NPI categories, 19 studies reported on measures aimed at reducing infection risk at individual level, 80 on measures to identify and isolate those who are or may become infectious, 71 on measures to reduce the numbers of contacts, 9 on measures to protect the most vulnerable, and 12 on travel and border restrictions. Forty-four studies reported on more than one NPI and were reported across multiple categories.

Nineteen of the 151 papers were preprints.

### Visual synthesis of results

**Fig. 2:**
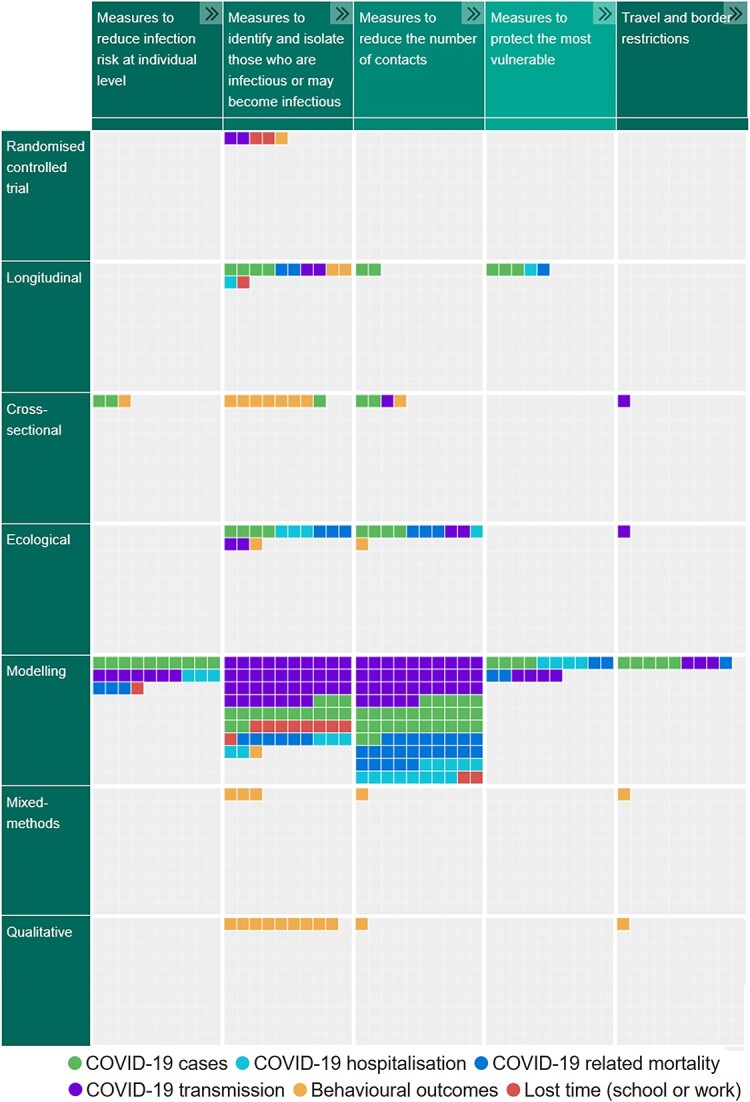
Screenshot of the interactive evidence gap map of studies reporting on the effectiveness of NPIs implemented in the community in the UK to reduce COVID-19 transmission.

The 151 studies identified were mapped onto an interactive evidence gap map available online.^49^ A screenshot of the map is presented in Fig. 2.

### Synthesis of results by NPI category

Table 2 shows the numbers of studies identified by NPI category and study design.

**Table 2:** Number of included studies by NPI category and study design

Study design	NPI category				
	Measures to reduce infection at individual level	Measures to identify and isolate those who are infectious or may become infectious	Measures to reduce the number of contacts	Measures to protect the most vulnerable	Travel and border restrictions
Randomized controlled trial	0	2	0	0	0
Non-randomized controlled trial	0	0	0	0	0
Longitudinal study	0	7	2	3	0
Cross-sectional study	3	7	3	0	1
Ecological study	0	6	7	0	1
Modelling study	16	46	57	6	8
Mixed-methods study	0	3	1	0	1
Qualitative study	0	9	1	0	1

**Measures to reduce infection at an individual level (19 studies):** face covering use was evaluated in 14 of the 19 studies, physical distancing in 7 studies, ventilation in 5 studies, hand hygiene in 2 studies and cleaning in one study. No evidence was identified on respiratory hygiene.

Sixteen studies were modelling and 3 were cross-sectional. No experimental or longitudinal studies were identified, suggesting that the studies available within this NPI category provide low level of evidence in terms of study design.

**Measures to identify and isolate those who are infectious or may become infectious (80 studies):** asymptomatic testing was reported in 30 of the 80 studies, contact tracing in 27 studies, isolation of cases in 21 studies, test and release strategies in 14 studies, isolation of contacts in 10 studies and symptomatic testing in 7 studies.

Of these 80 studies, there were 46 modelling, 2 RCTs, 7 longitudinal (although only one was prospective with a control group), 7 cross-sectional, 6 ecological, 3 mixed-methods and 9 qualitative.

This body of evidence provides slightly stronger evidence in terms of study design than for the other categories due to the presence of the RCTs and individual-level observational studies, although it remains mainly based on modelling, ecological, mixed-methods and qualitative studies.

**Measures to reduce the number of contacts (71 studies):** lockdown was the most commonly evaluated NPI in this category with 36 studies, followed by school closures in 17 studies, limitation of social contacts in 17 studies, school bubbles in 5 studies, workplace closure or work from home in 5 studies, tiered restrictions in 4 studies, restrictions of large gatherings in 3 studies, cohorting in 3 studies and hospitality setting closures in one study.

Of these 71 studies, there were 57 modelling, 2 prospective longitudinal, 3 cross-sectional, 7 ecological, one mixed-methods and one qualitative. No experimental studies were identified.

**Measures to protect the most vulnerable (9 studies):** 6 of the studies reporting on shielding were modelling and 3 were longitudinal. No experimental studies were identified.

**Travel restrictions and border measures (12 studies):** 7 studies reported on border measures and 5 on travel restrictions. Of the 12 studies, 8 were modelling, one cross-sectional, one ecological, one mixed-methods and one qualitative. No experimental or individual-level observational studies were identified.

## Discussion

### Main finding of this study

Our mapping review shows that most of the studies identified reported on measures to identify and isolate those who are or may become infectious, and on measures to reduce the number of contacts. Apart from face covering use, there was a lack of evidence for measures to reduce infection risk at individual level such as hand and respiratory hygiene, ventilation and cleaning.

Of the 151 studies, there were only 2 RCTs and 10 longitudinal observational studies, suggesting that despite the large number of papers published there is a lack of robust evaluation of the NPIs implemented during the pandemic in the UK.

### What is already known on this topic

Contact tracing, testing and isolation have been a regular part of public health efforts to control the spread of infection,^10^ although these strategies were never previously implemented at the scale or cost seen during the COVID-19 pandemic.^201^

Other NPIs such as cleaning surfaces or hand and respiratory hygiene have been used to reduce the spread of infectious diseases long before the COVID-19 pandemic, but there is still a lack of evidence on their effectiveness against COVID-19.^6^^–^^9^ For these measures, consideration of transmission mode is crucial. It is now well accepted that COVID-19 is airborne, and that long-distance airborne transmission can happen in indoor settings,^202^ which impacts on the type of measures required to mitigate transmission.^203^

There is evidence that the implementation of packages of NPIs were effective in controlling the COVID-19 pandemic before treatments and vaccination became available.^9^^,^^12^^,^^13^ However, whilst the number of studies reporting on effectiveness of individual NPIs accumulated during the pandemic,^8^^,^^204^^–^^206^ there are still uncertainties regarding their effectiveness. This is partly due to the challenges in interpreting the evidence arising from differences in methodologies between studies^14^ and differences in implementation between countries and settings.

### What this study adds

By identifying and categorizing studies evaluating the effectiveness of individual NPIs in the UK, this mapping review with evidence gap map^49^ allows a better understanding of the evidence generated during the pandemic to inform evaluation of interventions in the context of future pandemic preparedness.

We identified evidence gaps in the evaluation of measures to reduce infection at individual level in the UK, with the exception of face covering use. Although this could partly be because we excluded studies reporting on efficacy rather than effectiveness, our findings are in line with those from a recent systematic review by the Royal Society, which concluded that there was an evidence gap for surface disinfection and that the evidence available for ventilation was of low or very low quality and certainty.^6^ Therefore, the need remains to assess the effectiveness of hand and respiratory hygiene, ventilation and cleaning.

Only one study was identified on hospitality setting closures, although this could be due to the overlap between setting closures and lockdown. Limitation of social contacts can also sometimes overlap with lockdown, although we identified studies reporting on specific measures implemented in the UK such as Christmas household bubbles,^92^ formation of household support bubbles^95^ or social bubbles.^107^

Two-thirds of the studies identified were modelling. Models can be useful to assess the potential impact of different scenarios and inform policy, but they do not provide real-world evidence of effectiveness. In addition, modelling studies conducted during the COVID-19 pandemic were of varied methodological quality,^207^^,^^208^ although quality assessment was beyond the scope of this mapping review.

Only 2 RCTs were identified, both reporting on test and release strategies (daily testing with the aim to reduce cases and contacts earlier from isolation) implemented in England in 2021.^150^^,^^151^ One of the RCTs and its linked qualitative study were conducted in secondary schools,^151^^,^^195^ whilst the other RCT, also with a linked qualitative study, was conducted in the wider community.^150^^,^^194^ These RCTs were informed by proof-of-concept studies on acceptability and feasibility conducted in the general population^159^^,^^168^^,^^193^ and in key workers in Liverpool.^160^ These studies, which reported on transmission but also on behavioural outcomes and impact on school and work attendance, constitute a comprehensive body of evidence on evaluation of an intervention and provide the strongest evidence identified in this review in terms of study design and outcomes assessed.

Apart from these 2 RCTs and the modelling studies, the remaining studies were mainly ecological, cross-sectional and longitudinal, some without control groups or without before-after measurements, therefore providing low level of evidence in terms of study design.

Whilst acknowledging the wider challenges of undertaking research, especially RCTs, during the pandemic (including limited resources, feasibility and ethical considerations), the lack of experimental and analytical observational studies has limited researchers’ and policymakers’ ability to assess the interventions and policies implemented. This is a key learning point for future pandemic preparedness, and there is a need to build evaluation into the design and implementation of public health interventions and policies from the start of any future pandemic or other public health emergency.

To support clarity of messaging, future work on research and evaluation, and to likely aid the public’s acceptability and adherence to these measures, more descriptive terminologies such as ‘behavioural, social and cultural interventions’ (BSCI) or ‘public health and social measures’ (PHSM), which is used by the World Health Organization (WHO),^209^^,^^210^ are being considered instead of ‘non-pharmaceutical interventions’ (NPI).^211^

### Limitations of this study

As the review question was broad, pragmatic decisions were made to make the scope manageable. In particular, studies reporting solely on packages or stringency of measures were excluded, and relevant studies may have been missed. This exclusion criteria may also have led to potential inconsistencies as measures such as lockdown can be seen as a package of NPIs^212^ or as an individual NPI (e.g. if implemented as a government policy). As our focus was on NPIs implemented in the UK, we considered lockdown as an individual NPI and separated out other measures such as setting closures. Indeed in the UK, lockdown and setting closures were not always implemented simultaneously and, in addition, were sometimes implemented slightly differently across the UK.^1^^,^^213^

The main methodological limitation of our study is that we followed streamlined methodologies, such as full-text screening, coding and data extraction being done by one reviewer and checked by another rather than being done independently by 2 reviewers. Potential inconsistencies between reviewers were minimized through quality assurance processes.

## Conclusion

The findings from our rapid mapping review on effectiveness of NPIs implemented in community settings in the UK suggest that although large numbers of papers were published, there are still important gaps in evidence. Most of the research focused on measures aimed to identify and isolate infectious or potentially infectious individuals and to reduce the numbers of contacts. Except for face covering use, there is an evidence gap regarding effectiveness of NPIs implemented at individual level, particularly hand and respiratory hygiene, ventilation and cleaning.

Our results also show that the studies identified provide low level of evidence in terms of study design, with a lack of experimental and analytical observational studies. Our findings highlight the need to build evaluation into the design and implementation of public health interventions and policies, supporting ongoing work on building capabilities for evaluating interventions.

This mapping review will be followed by a series of rapid reviews to critically assess and synthesize the evidence identified. There is also a need to review the socioeconomic impact of NPIs, including the unintended consequences of NPIs on vulnerable groups and on health inequalities. UKHSA is currently working with academic partners to address these gaps.

## Supplementary Material

Supplementary_data_file_1_fdae025

Supplementary_data_file_2_fdae025

Supplementary_data_file_3_fdae025

Supplementary_data_file_4_fdae025

Supplementary_data_5_-_List_of_excluded_studies_fdae025

Supplementary_data_file_6_-_Coding_fdae025

## Notes

D. Duval and B. Evans are joint first authors

**D Duval**, Evidence Review Lead

**B Evans**, Senior Evidence Reviewer

**A Sanders**, Senior Evidence Reviewer

**J Hill**, Information Scientist

**A Simbo**, Senior Scientist

**T Kavoi**, Public Health Specialty Registrar

**I Lyell**, Public Health Specialty Registrar

**Z Simmons**, Senior Evidence Reviewer

**M Qureshi**, Evidence Reviewer

**N Pearce-Smith**, Senior Information Scientist

**CR Arevalo**, Deputy Director

**CR Beck**, Deputy Director

**R Bindra**, Deputy Director

**I Oliver**, Director General Science and Research and Chief Scientific Officer.
