# Supplementary material for: Non-pharmaceutical interventions to reduce COVID-19 transmission in the UK: a rapid mapping review and interactive evidence gap map
Source: J Public Health (Oxf). 2024 Feb 29;46(2):e279–93. doi: 10.1093/pubmed/fdae025 (PMC11141784; doi:10.1093/pubmed/fdae025)
Supplement: Supplementary_data_file_1_fdae025 [file supplementary_data_file_1_fdae025.docx]

## Supplementary Data 1. Modifications to the protocol

**Sources searched**

WHO COVID-19 Research Database was listed as a source in the protocol but due to the high number of hits retrieved by a trial search of this database, of which a large proportion were not specific to the UK, it was decided to not use the WHO COVID-19 Research Database and instead search CoronaCentral (which uses machine learning to identify and categorise published papers and preprints on SARS-CoV-2). Searching a limited number of databases is in line with rapid review methodologies.^17, 214^

**Screening**

It was anticipated that title and abstract screening would be done by 2 reviewers, with 10% duplicate; however, due to the high number of records retrieved by the literature search, 4 reviewers were involved in title and abstract screening and additional screening was done in duplicate (15% in total) to ensure consistency between reviewers.

**Coding**

Eligibility criteria for study design and NPI were listed in the protocol, but some modifications were made to their categorisation for the purpose of the mapping (none of these changes influenced eligibility of the studies):

- ‘isolation’ was split into ‘isolation of cases’ and ‘isolation of contacts’
- ‘limitations of social events’ was split into ‘limitation of social contacts’ and ‘restriction of large gatherings’
- ‘cohort study’ and ‘case control study’ were replaced by ‘longitudinal study’ to reflect whether a study had followed a group of participants over time. This is because some of the studies identified in this review were natural experiments which did not always fit easily into the conventional categories (RCTs, cohort studies, case control studies, and cross-sectional studies) and did not always provide analytical statistics, whether due to a lack of comparator group, a lack of pre-intervention measurement, or both.

**Data extraction**

The protocol reported that the key findings of the studies would be extracted. Due to the high volume of records identified, it was agreed between reviewers and topic advisors that this would not be done. Coding at the full-text screening stage was not part of the initial protocol; this was decided once title and abstract screening had been completed.
