# Supplementary material for: Non-pharmaceutical interventions to reduce COVID-19 transmission in the UK: a rapid mapping review and interactive evidence gap map
Source: J Public Health (Oxf). 2024 Feb 29;46(2):e279–93. doi: 10.1093/pubmed/fdae025 (PMC11141784; doi:10.1093/pubmed/fdae025)
Supplement: Supplementary_data_file_2_fdae025 [file supplementary_data_file_2_fdae025.docx]

## Supplementary Data 2. Inclusion and exclusion criteria

|  | Included | Excluded |
| --- | --- | --- |
| **Population/country** | All population, UK | Non-UK studies |
| **Settings** | Community settings | Health and social care settings |
| **Context** | COVID-19 pandemic | Other infectious diseases |
| **Intervention / exposure** | All types of NPI implemented in the UK, including:   - testing - contact tracing - isolation of cases - isolation of contacts - lockdown - physical distancing - limitation of social contact - limitation of large gathering - cleaning - hand and respiratory hygiene - face covering - school closures - workplace closure / working from home - hospitality setting closure - border restriction - ventilation     Studies that reported on the impact of not having the intervention (for instance, impact of delayed contact tracing due to faulty system, or impact of re-opening schools) were considered for inclusion | - Studies assessing performance of specific tests or products (rather than assessing an intervention) - Studies comparing effectiveness of different types of face coverings (rather than assessing effectiveness of wearing face coverings as an intervention) - PPE (gowns, goggles, aprons etc) and interventions specific to health and social care settings |
| **Outcomes** | Any outcomes related to the impact of the intervention on the COVID-19 pandemic, including but not limited to:   - COVID-19 transmission / cases - COVID-19 incidence / prevalence - COVID-19 hospitalisation - COVID-19 deaths - compliance, adherence, knowledge and behaviour - socioeconomic impacts | Studies were excluded if their primary outcomes are:   - economic outcomes - health outcomes other than those related to COVID-19 outcomes     Studies that reported on knowledge and behavioural factors related to COVID-19 but not as impact of an intervention were excluded. |
| **Language** | English |  |
| **Date of publication** | 1 January 2020 to February 2023 |  |
| **Study design** | - Experimental and quasi experimental studies - Cohort and case control studies - Outbreak investigations - Cross sectional studies - Ecological studies^a^ - Modelling studies^b^ | - Systematic or narrative reviews - Guidelines - Opinion pieces |
| **Publication type** | Peer-reviewed and preprint | Conference abstracts |
| ^a^ International ecological studies which did not provide evidence on effectiveness of NPI as implemented in the UK were excluded (for instance, cross-countries ecological studies that pooled UK data with data from other countries without reporting effectiveness data specific to the UK were excluded).  ^b^ Modelling studies that reported on hypothetical cases or populations not directly relevant to the UK were excluded (modelling studies using hypothetical cases/assumptions were included if authors were from the UK). | | |
