# Supplementary material for: Non-pharmaceutical interventions to reduce COVID-19 transmission in the UK: a rapid mapping review and interactive evidence gap map
Source: J Public Health (Oxf). 2024 Feb 29;46(2):e279–93. doi: 10.1093/pubmed/fdae025 (PMC11141784; doi:10.1093/pubmed/fdae025)
Supplement: Supplementary_data_file_3_fdae025 [file supplementary_data_file_3_fdae025.docx]

## Supplementary Data 3. Database search strategies

Ovid Medline ALL (1946 to 28 February 2023)

| 1. exp SARS-CoV-2/ (149010) |
| --- |
| 1. exp COVID-19/ (211700) |
| 1. (corona* adj1 (virus* or viral*)).tw,kw,kf. (6005) |
| 1. (CoV not (Coefficien* or "co-efficien*" or covalent* or Covington* or covariant* or covarianc* or "cut-off value*" or "cutoff value*" or "cut-off volume*" or "cutoff volume*" or "combined optimi?ation value*" or "central vessel trunk*" or CoVR or CoVS)).tw,kw,kf. (114761) |
| 1. (coronavirus* or 2019nCoV* or 19nCoV* or "2019 novel*" or Ncov* or "n-cov" or "SARS-CoV-2*" or "SARSCoV-2*" or SARSCoV2* or "SARS-CoV2*" or "severe acute respiratory syndrome*" or COVID*2).tw,kw,kf. (348974) |
| 1. exp COVID-19 Vaccines/ (19725) |
| 1. exp COVID-19 Testing/ (10582) |
| 1. or/1-7 (356560) |
| 1. Contact Tracing/ (6149) |
| 1. ((contact or source or infection or patient or case) adj2 (screen* or notificat* or trac* or investig*)).tw,kf. (84268) |
| 1. (NPI* or ((non-pharm* or nonpharm*) adj intervention*) or "public health measure*" or (prevent* adj2 measure*)).tw,kf. (64487) |
| 1. 9 or 10 or 11 (151494) |
| 1. Quarantine/ (6157) |
| 1. Social Isolation/ (16021) |
| 1. (quarantin* or isolat*).ti,kf. or (quarantin* or isolat*).ab. /freq=2 (660072) |
| 1. 13 or 14 or 15 (674277) |
| 1. exp *COVID-19 Testing/ (2922) |
| 1. Point-of-Care Testing/ or Self-Testing/ (4286) |
| 1. Reagent Kits, Diagnostic/ (17583) |
| 1. ("lateral flow" or LFT or LFA or LFD or LFIA).tw,kf. (14022) |
| 1. ((COVID or Corona*) adj2 test*).tw,kf. (6976) |
| 1. 17 or 18 or 19 or 20 or 21 (44206) |
| 1. (lockdown* or lock-down*).ti,kf. or (lockdown* or lock-down*).ab. /freq=2 (11563) |
| 1. ((stay adj2 home) or shielding).tw,kf. (18972) |
| 1. Physical Distancing/ (2290) |
| 1. ((physical* or social*) adj distan*).tw,kf. (14129) |
| 1. ((social* or societal* or gathering* or meeting* or event*) adj3 (restrict* or prohibit* or limit* or ban* or cancel*)).tw,kf. (13637) |
| 1. (tier* adj2 restric*).tw,kf. (26) |
| 1. 23 or 24 or 25 or 26 or 27 or 28 (55787) |
| 1. exp Disinfectants/ or Disinfection/ (87461) |
| 1. ((environment* or surface* or home* or house* or workplace*) adj3 disinfect*).tw,kf. (2948) |
| 1. ((environment* or surface* or home* or house* or workplace*) adj3 clean*).tw,kf. (7988) |
| 1. ((environment* or surface* or home* or house* or workplace*) adj3 decontaminat*).tw,kf. (1294) |
| 1. deep clean*.tw,kf. (69) |
| 1. 30 or 31 or 32 or 33 or 34 (97245) |
| 1. exp Hand Hygiene/ (8077) |
| 1. (hand wash* or handwash*).tw,kf. (6450) |
| 1. hand saniti*.tw,kf. (961) |
| 1. ((hand or personal) adj hygiene).tw,kf. (8768) |
| 1. 36 or 37 or 38 or 39 (17443) |
| 1. (facemask* or mask*).tw,kf. (100303) |
| 1. Masks/ (7178) |
| 1. (face adj2 (cover* or protect*)).tw,kf. (1081) |
| 1. (mouth adj2 (cover* or protect*)).tw,kf. (361) |
| 1. (nose adj2 (cover* or protect*)).tw,kf. (152) |
| 1. (respirator or respirators).tw,kf. (6906) |
| 1. 41 or 42 or 43 or 44 or 45 or 46 (108178) |
| 1. ((universit* or college* or school*) adj3 clos*).tw,kf. (3072) |
| 1. ((office* or work*) adj3 clos*).tw,kf. (5675) |
| 1. (home* adj3 work*).tw,kf. (10428) |
| 1. ((hospitalit* or restaurant* or cafe* or venue* or shop* or retail* or hotel* or leisure or gym* or cinema* or theatre* or theater*) adj3 clos*).tw,kf. (486) |
| 1. 48 or 49 or 50 or 51 (19309) |
| 1. border health.tw,kf. (266) |
| 1. (Travel* adj5 (ban* or restrict*)).tw,kf. (1827) |
| 1. (Border* adj5 (control* or restrict*)).tw,kf. (2125) |
| 1. ((entrance or Entry) adj5 restrict*).tw,kf. (1034) |
| 1. (Movement* adj5 restrict*).tw,kf. (5642) |
| 1. 53 or 54 or 55 or 56 or 57 (10676) |
| 1. ((ventilation or ventilated) and (transmission* or distanc* or dispers* or aerosol* or airborne or air qualit* or indoor air)).tw,kf. (8989) |
| 1. Ventilation/ (6327) |
| 1. (air flow* or airflow* or aero* or air condition*).tw,kf. (37245) |
| 1. Air Conditioning/ (2867) |
| 1. (air filter* or air purif* or air filtration).tw,kf. (2748) |
| 1. Air Filters/ (586) |
| 1. (air chang* or air exchang*).tw,kf. (1852) |
| 1. (air adj3 (recondition* or re condition*)).tw,kf. (1) |
| 1. (air adj3 replac*).tw,kf. (400) |
| 1. (indoor air adj3 qualit*).tw,kf. (2918) |
| 1. HVAC.tw,kf. (564) |
| 1. Air Microbiology/ (8274) |
| 1. 59 or 60 or 61 or 62 or 63 or 64 or 65 or 66 or 67 or 68 or 69 or 70 (62693) |
| 1. 12 or 16 or 22 or 29 or 35 or 40 or 47 or 52 or 58 or 71 (1195929) |
| 1. 8 and 72 (63507) |
| 1. exp United Kingdom/ (388488) |
| 1. (national health service* or nhs*).ti,ab,in. (262664) |
| 1. (english not ((published or publication* or translat* or written or language* or speak* or literature or citation*) adj5 english)).ti,ab. (48148) |
| 1. (gb or "g.b." or britain* or (british* not "british columbia") or uk or "u.k." or united kingdom* or (england* not "new england") or northern ireland* or northern irish* or scotland* or scottish* or ((wales or "south wales") not "new south wales") or welsh*).ti,ab,jw,in. (2404695) |
| 1. (bath or "bath's" or ((birmingham not alabama*) or ("birmingham's" not alabama*) or bradford or "bradford's" or brighton or "brighton's" or bristol or "bristol's" or carlisle* or "carlisle's" or (cambridge not (massachusetts* or boston* or harvard*)) or ("cambridge's" not (massachusetts* or boston* or harvard*)) or (canterbury not zealand*) or ("canterbury's" not zealand*) or chelmsford or "chelmsford's" or chester or "chester's" or chichester or "chichester's" or coventry or "coventry's" or derby or "derby's" or (durham not (carolina* or nc)) or ("durham's" not (carolina* or nc)) or ely or "ely's" or exeter or "exeter's" or gloucester or "gloucester's" or hereford or "hereford's" or hull or "hull's" or lancaster or "lancaster's" or leeds* or leicester or "leicester's" or (lincoln not nebraska*) or ("lincoln's" not nebraska*) or (liverpool not (new south wales* or nsw)) or ("liverpool's" not (new south wales* or nsw)) or ((london not (ontario* or ont or toronto*)) or ("london's" not (ontario* or ont or toronto*)) or manchester or "manchester's" or (newcastle not (new south wales* or nsw)) or ("newcastle's" not (new south wales* or nsw)) or norwich or "norwich's" or nottingham or "nottingham's" or oxford or "oxford's" or peterborough or "peterborough's" or plymouth or "plymouth's" or portsmouth or "portsmouth's" or preston or "preston's" or ripon or "ripon's" or salford or "salford's" or salisbury or "salisbury's" or sheffield or "sheffield's" or southampton or "southampton's" or st albans or stoke or "stoke's" or sunderland or "sunderland's" or truro or "truro's" or wakefield or "wakefield's" or wells or westminster or "westminster's" or winchester or "winchester's" or wolverhampton or "wolverhampton's" or |
| 1. (worcester not (massachusetts* or boston* or harvard*)) or ("worcester's" not (massachusetts* or boston* or harvard*)) or (york not ("new york*" or ny or ontario* or ont or toronto*)) or ("york's" not ("new york*" or ny or ontario* or ont or toronto*))))).ti,ab,in. (1706641) |
| 1. (bangor or "bangor's" or cardiff or "cardiff's" or newport or "newport's" or st asaph or "st asaph's" or st davids or swansea or "swansea's").ti,ab,in. (68546) |
| 1. (aberdeen or "aberdeen's" or dundee or "dundee's" or edinburgh or "edinburgh's" or glasgow or "glasgow's" or inverness or (perth not australia*) or ("perth's" not australia*) or stirling or "stirling's").ti,ab,in. (251449) |
| 1. (armagh or "armagh's" or belfast or "belfast's" or lisburn or "lisburn's" or londonderry or "londonderry's" or derry or "derry's" or newry or "newry's").ti,ab,in. (32893) |
| 1. or/74-81 (3018015) |
| 1. (exp africa/ or exp americas/ or exp antarctic regions/ or exp arctic regions/ or exp asia/ or exp australia/ or exp oceania/) not (exp United Kingdom/ or europe/) (3293714) |
| 1. 82 not 83 (2857491) |
| 1. 73 and 84 (8515) |
| 1. limit 85 to yr="2021 -Current" (6488) |
| 1. limit 85 to dt=20210101-20230301 (6162) |
| 1. 86 or 87 (6630) |
| 1. exp animals/ not humans.sh. (5098481) |
| 1. 88 not 89 (6615) |

Embase (1974 to 2023 February 28)

| 1 exp severe acute respiratory syndrome coronavirus 2/ (94229) |
| --- |
| 2 coronavirus disease 2019/ (334121) |
| 3 experimental coronavirus disease 2019/ (19) |
| 4 (corona* adj1 (virus* or viral*)).tw,kw. (6696) |
| 5 (CoV not (Coefficien* or co-efficien* or covalent* or covington or covariant* or covarianc* or "cut-off value*" or "cutoff value*" or "cut-off volume*" or "cutoff volume*" or "combined optimi?ation value*" or "central vessel trunk" or CoVR or CoVS)).tw,kw. (125587) |
| 6 (coronavirus* or 2019nCoV* or 19nCoV* or "2019 novel*" or Ncov* or "n-cov" or "SARS CoV-2*" or "SARSCoV-2*" or SARSCoV2* or "SARS-CoV2*" or "severe acute respiratory syndrome*" or COVID*2).tw,kw. (420622) |
| 7 COVID-19 Testing/ (6989) |
| 8 exp SARS-CoV-2 vaccine/ (32697) |
| 9 or/1-8 (449718) |
| 10 exp contact examination/ (8433) |
| 11 ((contact or source or infection or patient or case) adj2 (screen* or notificat* or trac* or investig*)).tw,kf. (126880) |
| 12 (NPI* or ((non-pharm* or nonpharm*) adj intervention*)).tw,kf. (17727) |
| 13 10 or 11 or 12 (149110) |
| 14 exp *quarantine/ (2260) |
| 15 *social isolation/ (7766) |
| 16 (quarantin* or isolat*).ti,kf. or (quarantin* or isolat*).ab. /freq=2 (765061) |
| 17 14 or 15 or 16 (769152) |
| 18 *COVID-19 Testing/ (2681) |
| 19 exp *"point of care testing"/ (7577) |
| 20 self-testing/ (888) |
| 21 exp *infectious disease test kit/ (6296) |
| 22 ("lateral flow" or LFT or LFA or LFD or LFIA).tw,kf. (19510) |
| 23 lateral flow immunochromatography/ (1666) |
| 24 ((COVID or Corona*) adj2 test*).tw,kf. (9814) |
| 25 18 or 19 or 20 or 21 or 22 or 23 or 24 (44590) |
| 26 (lockdown* or lock-down*).ti,kf. or (lockdown* or lock-down*).ab. /freq=2 (12005) |
| 27 ((stay adj2 home) or shielding).tw,kf. (22223) |
| 28 *social distancing/ (1241) |
| 29 mass gathering/ (255) |
| 30 ((physical* or social*) adj distan*).tw,kf. (15382) |
| 31 ((social* or societal* or gathering* or meeting* or event*) adj3 (restrict* or prohibit* or limit* or ban* or cancel*)).tw,kf. (19180) |
| 32 (tier* adj2 restric*).tw,kf. (31) |
| 33 26 or 27 or 28 or 29 or 30 or 31 or 32 (65641) |
| 34 exp *disinfectant agent/ (203693) |
| 35 *disinfection/ (13092) |
| 36 *cleaning/ (3806) |
| 37 ((environment* or surface* or home* or house* or workplace*) adj3 disinfect*).tw,kf. (3528) |
| 38 ((environment* or surface* or home* or house* or workplace*) adj3 clean*).tw,kf. (9606) |
| 39 ((environment* or surface* or home* or house* or workplace*) adj3 decontaminat*).tw,kf. (1468) |
| 40 deep clean*.tw,kf. (141) |
| 41 34 or 35 or 36 or 37 or 38 or 39 or 40 (228536) |
| 42 exp *hand washing/ (5340) |
| 43 (hand wash* or handwash*).tw,kf. (8430) |
| 44 hand saniti*.tw,kf. (1308) |
| 45 ((hand or personal) adj hygiene).tw,kf. (13154) |
| 46 exp *personal hygiene/ (10419) |
| 47 42 or 43 or 44 or 45 or 46 (31536) |
| 48 (facemask* or mask*).tw,kf. (126775) |
| 49 exp *face mask/ or exp filtering facepiece respirator/ (5460) |
| 50 (face adj2 (cover* or protect*)).tw,kf. (1181) |
| 51 (mouth adj2 (cover* or protect*)).tw,kf. (379) |
| 52 (nose adj2 (cover* or protect*)).tw,kf. (202) |
| 53 (respirator or respirators).tw,kf. (6994) |
| 54 48 or 49 or 50 or 51 or 52 or 53 (134596) |
| 55 ((universit* or college* or school*) adj3 clos*).tw,kf. (3282) |
| 56 ((office* or work*) adj3 clos*).tw,kf. (8331) |
| 57 (home* adj3 work*).tw,kf. (13099) |
| 58 ((hospitalit* or restaurant* or cafe* or venue* or shop* or retail* or hotel* or leisure or gym* or cinema* or theatre* or theater*) adj3 clos*).tw,kf. (566) |
| 59 school closure/ (498) |
| 60 55 or 56 or 57 or 58 or 59 (24996) |
| 61 border health.tw,kf. (345) |
| 62 travel restriction/ (1004) |
| 63 (Travel* adj5 (ban* or restrict*)).tw,kf. (2013) |
| 64 (Border* adj5 (control* or restrict*)).tw,kf. (2622) |
| 65 ((entrance or Entry) adj5 restrict*).tw,kf. (1293) |
| 66 (Movement* adj5 restrict*).tw,kf. (7108) |
| 67 61 or 62 or 63 or 64 or 65 or 66 (13741) |
| 68 ((ventilation or ventilated) and (transmission* or distanc* or dispers* or aerosol* or airborne or air qualit* or indoor air)).tw,kf. (13101) |
| 69 *air conditioning/ (5878) |
| 70 (air flow* or airflow* or aerodynamic* or air condition*).tw,kf. (51380) |
| 71 (air filter* or air purif* or air filtration).tw,kf. (3368) |
| 72 exp air filter/ (2340) |
| 73 (air chang* or air exchang*).tw,kf. (2626) |
| 74 (air adj3 (recondition* or re condition*)).tw,kf. (4) |
| 75 (air adj3 replac*).tw,kf. (471) |
| 76 (indoor air adj3 qualit*).tw,kf. (4180) |
| 77 HVAC.tw,kf. (748) |
| 78 airborne virus/ (378) |
| 79 exp airborne transmission/ (1326) |
| 80 68 or 69 or 70 or 71 or 72 or 73 or 74 or 75 or 76 or 77 or 78 or 79 (76919) |
| 81 13 or 17 or 25 or 33 or 41 or 47 or 54 or 60 or 67 or 80 (1490125) |
| 82 9 and 81 (69831) |
| 83 exp United Kingdom/ (459099) |
| 84 (national health service* or nhs*).ti,ab,in,ad. (464290) |
| 85 (english not ((published or publication* or translat* or written or language* or speak* or literature or citation*) adj5 english)).ti,ab. (58584) |
| 86 (gb or "g.b." or britain* or (british* not "british columbia") or uk or "u.k." or united kingdom* or (england* not "new england") or northern ireland* or northern irish* or scotland* or scottish* or ((wales or "south wales") not "new south wales") or welsh*).ti,ab,jx,in,ad. (3704016) |
| 87 (bath or "bath's" or ((birmingham not alabama*) or ("birmingham's" not alabama*) or bradford or "bradford's" or brighton or "brighton's" or bristol or "bristol's" or carlisle* or "carlisle's" or (cambridge not (massachusetts* or boston* or harvard*)) or ("cambridge's" not (massachusetts* or boston* or harvard*)) or (canterbury not zealand*) or ("canterbury's" not zealand*) or chelmsford or "chelmsford's" or chester or "chester's" or chichester or "chichester's" or coventry or "coventry's" or derby or "derby's" or (durham not (carolina* or nc)) or ("durham's" not (carolina* or nc)) or ely or "ely's" or exeter or "exeter's" or gloucester or "gloucester's" or hereford or "hereford's" or hull or "hull's" or lancaster or "lancaster's" or leeds* or leicester or "leicester's" or (lincoln not nebraska*) or ("lincoln's" not nebraska*) or (liverpool not (new south wales* or nsw)) or ("liverpool's" not (new south wales* or nsw)) or ((london not (ontario* or ont or toronto*)) or ("london's" not (ontario* or ont or toronto*)) or manchester or "manchester's" or (newcastle not (new south wales* or nsw)) or ("newcastle's" not (new south wales* or nsw)) or norwich or "norwich's" or nottingham or "nottingham's" or oxford or "oxford's" or peterborough or "peterborough's" or plymouth or "plymouth's" or portsmouth or "portsmouth's" or preston or "preston's" or ripon or "ripon's" or salford or "salford's" or salisbury or "salisbury's" or sheffield or "sheffield's" or southampton or "southampton's" or st albans or stoke or "stoke's" or sunderland or "sunderland's" or truro or "truro's" or wakefield or "wakefield's" or wells or westminster or "westminster's" or winchester or "winchester's" or wolverhampton or "wolverhampton's" or (worcester not (massachusetts* or boston* or harvard*)) or ("worcester's" not (massachusetts* or boston* or harvard*)) or (york not ("new york*" or ny or ontario* or ont or toronto*)) or ("york's" not ("new york*" or ny or ontario* or ont or toronto*))))).ti,ab,in,ad. (2886585) |
| 88 (bangor or "bangor's" or cardiff or "cardiff's" or newport or "newport's" or st asaph or "st asaph's" or st davids or swansea or "swansea's").ti,ab,in,ad. (118676) |
| 89 (aberdeen or "aberdeen's" or dundee or "dundee's" or edinburgh or "edinburgh's" or glasgow or "glasgow's" or inverness or (perth not australia*) or ("perth's" not australia*) or stirling or "stirling's").ti,ab,in,ad. (396819) |
| 90 (armagh or "armagh's" or belfast or "belfast's" or lisburn or "lisburn's" or londonderry or "londonderry's" or derry or "derry's" or newry or "newry's").ti,ab,in,ad. (55082) |
| 91 or/83-90 (4522510) |
| 92 ((exp "arctic/ and antarctic"/) or exp oceanic regions/ or exp western hemisphere/ or exp africa/ or exp asia/) not (exp united kingdom/ or europe/) (3462654) |
| 93 91 not 92 (4270501) |
| 94 82 and 93 (10129) |
| 95 limit 94 to dc=20210101-20230301 (8225) |
| 96 limit 94 to yr="2021 -Current" (7711) |
| 97 95 or 96 (8285) |
| 98 limit 97 to conference abstracts (1979) |
| 99 97 not 98 (6306) |
| 100 (exp animal/ or animal experiment/ or nonhuman/) not (exp human/ or human experiment/) (7198958) |
| 101 99 not 100 (6125) |

NIH Covid-19 Portfolio: <https://icite.od.nih.gov/covid19/search/>

Date of searches: 01/03/2023

Searches in Covid 19 Portfolio were run separately for each NPI, and limited by source to ArXiv, MedrXiv and Research Square. No date limit was applied during search, but following download into EndNote all results with a publication year of 2020 were removed (according with criteria for date limit set out in protocol). Only results with publication years 2021, 2022 or 2023 went forward to duplicate removal against results from other databases and then title and abstract screening.

Duplicate removal

Duplicate search across all preprints was carried out in EndNote, separately to results from Medline and Embase searches.

4,739 duplicates were removed.

3,915 2020 publications were removed.

3,068 publications from 2021, 2022 and 2023 went forward for title and abstract screening. These results were copied into an EndNote library containing results from other sources searched (Medline: 6,615 results, Embase: 6,125 results and Corona Central: 38 results; NIH Preprint results: 3,068; total: 15,846 results).

| **Search terms** | **Preprint server** | **No. results** |
| --- | --- | --- |
| “contact tracing” OR “contact investigation” OR “contact screening” OR “contact investigation” OR “contact testing” | ArXiv | 258 |
|  | MedrXiv | 699 |
|  | Research Square | 296 |
| (quarantin* or isolat*) OR (lockdown* or lock-down* OR shielding) | ArXiv | 178 |
|  | MedrXiv | 1,295 |
|  | Research Square | 1250 |
| ("lateral flow" OR “COVID test” OR ”Coronavirus test” OR “home test”) | ArXiv | 12 |
|  | MedrXiv | 332 |
|  | Research Square | 101 |
| “physical distancing” OR “social distancing” OR “movement restriction” OR “mass gathering” OR “tiered restriction” | ArXiv | 446 |
|  | MedrXiv | 1,593 |
|  | Research Square | 983 |
| “environmental disinfection”~3 OR “surface disinfection”~3 OR “home disinfection”~3 OR “house disinfection”~3 OR “workplace disinfection”~3 OR deep clean* | ArXiv | 36 |
|  | MedrXiv | 475 |
|  | Research Square | 459 |
| “environmental cleaning”~3 OR “surface cleaning”~3 OR “home cleaning”~3 OR “house cleaning”~3 OR “workplace cleaning”~3 OR deep clean* | ArXiv | 36 |
|  | MedrXiv | 475 |
|  | Research Square | 459 |
| "hand santizer" OR "hand santiser" OR "hand washing" OR "hand hygiene" or handwashing | ArXiv | 2 |
|  | MedrXiv | 76 |
|  | Research Square | 73 |
| facemask or mask OR face covering | ArXiv | 4 |
|  | MedrXiv | 87 |
|  | Research Square | 83 |
| ("school closure" OR "workplace closure" OR "hospitality closure"~3 OR "home working") | ArXiv | 29 |
|  | MedrXiv | 258 |
|  | Research Square | 149 |
| “border health” OR “travel ban” OR “travel restriction” | ArXiv | 42 |
|  | MedrXiv | 236 |
|  | Research Square | 197 |
| (airflow* or “air flow” OR “air quality” or “indoor air” OR “air conditioning” OR “air filtration” OR HVAC) | ArXiv | 11 |
|  | MedrXiv | 50 |
|  | Research Square | 35 |
| “non pharmaceutical intervention” | ArXiv | 142 |
|  | MedrXiv | 655 |
|  | Research Square | 210 |

CoronaCentral search

<https://coronacentral.ai/>

Date of search: 02 March 2023

Searched for ‘NPI’ – filtered to virus type ‘Sars-Cov-2’ and Location: UK, Bristol and Oxford (only UK locations available)
