# Supplementary material for: Non-pharmaceutical interventions to reduce COVID-19 transmission in the UK: a rapid mapping review and interactive evidence gap map
Source: J Public Health (Oxf). 2024 Feb 29;46(2):e279–93. doi: 10.1093/pubmed/fdae025 (PMC11141784; doi:10.1093/pubmed/fdae025)
Supplement: Supplementary_data_file_4_fdae025 [file supplementary_data_file_4_fdae025.docx]

## Supplementary Data 4. List of codes for the evidence gap map

For data charting of NPIs, we used 2 levels of coding (parent and child) to organise individual NPIs within 5 categories:

- measures to reduce infection risk at individual level
- face covering use
- physical distancing (that is, keeping a distance of 1 or 2 metres between people, sometimes called ‘social distancing’)
- hand and respiratory hygiene (but mapped as ‘hand hygiene’ only as no evidence on respiratory hygiene was identified)
- cleaning (such as cleaning of surfaces)
- ventilation
- measures to identify and isolate those who are infectious or may become infectious
- contact tracing
- asymptomatic testing
- symptomatic testing
- isolation of cases
- isolation of contacts
- test and release strategies
- measures to reduce the numbers of contacts
- lockdown
- tiered restrictions
- hospitality setting closures
- workplace closure or working from home
- school closures
- school bubbles
- cohorting
- limitation of social contacts
- restrictions of large gatherings
- measures to protect the most vulnerable
- shielding measures
- travel and border restrictions
- border measures (such as screening, self-isolation or testing on arrival in the UK)
- travel restrictions (such as travel corridors and restrictions on international or regional travel)

For outcomes, the following codes were used (one level of coding):

- COVID-19 transmission (such as R numbers and secondary infection rates, but proxy data for transmission risk such as whether an outbreak happened was also included)
- COVID-19 cases (such as number of positive tests)
- COVID-19 hospitalisations* (such as number of hospitalisations or Intensive Care Unit admissions)
- COVID-19 mortality* (such as number of COVID-19 related deaths reported by the Office for National Statistics)
- behavioural outcomes (such as compliance, adherence, perceptions and attitudes)
- lost time (school or work) as a measure of how the different NPI implemented impacted school or work attendance

*included when used as proxies for transmission

For study designs, the following codes were used (one level of coding):

- randomised controlled trials
- non randomised controlled trials (no study identified)
- longitudinal studies
- cross-sectional studies
- ecological studies
- modelling studies
- mixed-methods studies
- qualitative studies
