# Supplementary material for: Non-pharmaceutical interventions to reduce COVID-19 transmission in the UK: a rapid mapping review and interactive evidence gap map
Source: J Public Health (Oxf). 2024 Feb 29;46(2):e279–93. doi: 10.1093/pubmed/fdae025 (PMC11141784; doi:10.1093/pubmed/fdae025)
Supplement: Supplementary_data_5_-_List_of_excluded_studies_fdae025 [file supplementary_data_5_-_list_of_excluded_studies_fdae025.docx]

Supplementary Data 5. List of excluded studies

| **Author** | **Year** | **Title** |
| --- | --- | --- |
| **Not UK n=86** | | |
| Abbey and others | 2022 | Exploring the effects of activity-preserving time dilation on the dynamic interplay of airborne contagion processes and temporal networks using an interaction-driven model |
| Aganovic and others | 2022 | Modeling the impact of indoor relative humidity on the infection risk of five respiratory airborne viruses |
| Ahmed and others | 2021 | Mechanistic modelling of COVID-19 and the impact of lockdowns on a short-time scale |
| Amuedo-Dorantes and others | 2021 | Early adoption of non-pharmaceutical interventions and COVID-19 mortality |
| Ashcroft and others | 2022 | Test-trace-isolate-quarantine (TTIQ) intervention strategies after symptomatic COVID-19 case identification |
| Bandyopadhyay and others | 2021 | Learning versus habit formation: Optimal timing of lockdown for disease containment |
| Bartolucci and others | 2022 | How distant? An experimental analysis of students' COVID-19 exposure and physical distancing in university buildings |
| Bhoi and others | 2021 | Communicable Disease Pandemic: A Simulation Model Based on Community Transmission and Social Distancing |
| Biala and others | 2021 | How Efficient is Contact Tracing in Mitigating the Spread of Covid-19? A Mathematical Modeling Approach |
| Bongiorno and others | 2021 | A multi-layer network model to assess school opening policies during the COVID-19 vaccination campaign |
| Bonnet and others | 2023 | Is there a role for RDTs as we live with COVID-19? An assessment of different strategies |
| Boyer and others | 2022 | Infectious disease dynamics and restrictions on social gathering size |
| Burman and others | 2021 | A Flexible Agent-Based Model to Study COVID-19 Outbreak -- A Generic Approach |
| Cao and others | 2021 | A COVID-19 Spread Model of the Discrete Grid to Assess the Potential of Non-pharmaceutical Interventions |
| Chowdhury and others | 2022 | Incorporating the mutational landscape of SARS-COV-2 variants and case-dependent vaccination rates into epidemic models |
| Chowell and others | 2021 | Harnessing testing strategies and public health measures to avert COVID-19 outbreaks during ocean cruises |
| Cotman and others | 2021 | Factors affecting aerosol SARS-CoV-2 transmission via HVAC systems; a modeling study |
| Daghriri and others | 2021 | Quantifying the effects of social distancing on the spread of covid-19 |
| Das and others | 2021 | COVID-19: Analytic results for a modified SEIR model and comparison of different intervention strategies |
| Dave and others | 2021 | When Do Shelter-in-Place Orders Fight Covid-19 Best? Policy Heterogeneity across States and Adoption Time |
| David and others | 2021 | Assessing COVID prevention strategies to permit the safe opening of college campuses in fall 2021 |
| Duives and others | 2021 | The multi-dimensional challenges of controlling SARS-CoV-2 transmission in indoor spaces: Insights from the linkage of a microscopic pedestrian simulation and virus transmission models |
| Egeren and others | 2022 | No magic bullet: limiting in-school transmission in the face of variable SARS-CoV-2 viral loads |
| Endo and others | 2022 | Simulating respiratory disease transmission within and between classrooms to assess pandemic management strategies at schools |
| Favero and others | 2022 | Modelling preventive measures and their effect on generation times in emerging epidemics |
| Foncea and others | 2021 | Optimal Testing Strategies to Monitor COVID-19 Traced Contacts |
| Foncea and others | 2022 | Replacing quarantine of COVID-19 contacts with periodic testing is also effective in mitigating the risk of transmission |
| Frieswijk and others | 2021 | Modelling the Effect of Vaccination and Human Behaviour on the Spread of Epidemic Diseases on Temporal Networks |
| Gandolfi and others | 2022 | A new threshold reveals the uncertainty about the effect of school opening on diffusion of Covid-19 |
| Gazquez and others | 2021 | Poster as Tool to Improve Hand Hygiene Among Health Science Students: Case-control Study |
| Godoy and others | 2023 | Implementation and spillovers of local non-pharmaceutical interventions |
| Goldsztejn and others | 2020 | Public policy and economic dynamics of COVID-19 spread: A mathematical modeling study |
| Grimm and others | 2021 | Extensions of the SEIR model for the analysis of tailored social distancing and tracing approaches to cope with COVID-19 |
| Gugole and others | 2021 | Uncertainty quantification and sensitivity analysis of COVID-19 exit strategies in an individual-based transmission model |
| Gunaratne and others | 2021 | Evaluating Efficacy of Indoor Non-Pharmaceutical Interventions against COVID-19 Outbreaks with a Coupled Spatial-SIR Agent-Based Simulation Framework |
| Han and others | 2021 | Modeling of suppression and mitigation interventions in the COVID-19 epidemics |
| Harmon and others | 2022 | The Facility Infection Risk EstimatorTM: A web application tool for comparing indoor risk mitigation strategies by estimating airborne transmission risk |
| Hasan and others | 2022 | Data-driven modeling and forecasting of COVID-19 outbreak for public policy making |
| Huang and others | 2022 | Airborne transmission of the Delta variant of SARS-CoV-2 in an auditorium |
| James and others | 2021 | Successful contact tracing systems for COVID-19 rely on effective quarantine and isolation |
| Jin and others | 2022 | A SEIRD+V Model for the Effect of Vaccination and Social Distancing on SARS-CoV-2 Infection and Mortality |
| Johnson and others | 2022 | Robust models of SARS-CoV-2 heterogeneity and control |
| Kearney and others | 2022 | Compliance with local travel restrictions and face masks during first phase of COVID-19 pandemic in Ireland: a national survey |
| Kennedy and others | 2021 | Modeling aerosol transmission of SARS-CoV-2 in multi-room facility |
| Khatami and others | 2022 | Deep reinforcement learning framework for controlling infectious disease outbreaks in the context of multi-jurisdictions |
| Kiang and others | 2021 | Routine asymptomatic testing strategies for airline travel during the COVID-19 pandemic: a simulation study |
| Klimek and others | 2022 | Small coverage effect in epidemic network models shows that masks can become more effective with less people wearing them |
| Kotil | 2021 | Emergent effects of contact tracing robustly stabilize outbreaks |
| Kuhfeldt and others | 2022 | Minimal SARS-CoV-2 classroom transmission at a large urban university experiencing repeated into campus introduction |
| Kurnitski and others | 2021 | Respiratory infection risk-based ventilation design method |
| Leon and others | 2021 | Nonpharmaceutical Interventions Remain Essential to Reducing COVID-19 Burden Even in a Well-Vaccinated Society: A Modeling Study |
| Liu and others | 2021 | Continuous Learning and Inference of Individual Probability of SARS-CoV-2 Infection Based on Interaction Data |
| Mahmood and others | 2021 | Contextual Contact Tracing based on Stochastic Compartment Modeling and Spatial Risk Assessment |
| Maiorana and others | 2021 | Effectiveness of isolation measures with app support to contain COVID-19 epidemics: a parametric approach |
| Mairanowski and others | 2021 | Functional dependence of COVID-19 growth rate on lockdown conditions and rate of vaccination |
| Majeed and others | 2022 | Variant-specific interventions to slow down replacement and prevent outbreaks |
| Mancastroppa and others | 2022 | Sideward contact tracing and the control of epidemics in large gatherings |
| McAloon and others | 2022 | Potential application of Rapid Antigen Diagnostic Tests for the detection of infectious individuals attending mass gatherings - a simulation study |
| McGee and others | 2021 | Model-driven mitigation measures for reopening schools during the COVID-19 pandemic |
| Mendoza and others | 2021 | Implementation of a pooled surveillance testing program for asymptomatic SARS-CoV-2 infections in K-12 schools and universities |
| Mohammadi and others | 2022 | Human behaviour, NPI and mobility reduction effects on COVID-19 transmission in different countries of the world |
| Moritz and others | 2021 | The risk of indoor sports and culture events for the transmission of COVID-19 |
| Pang and others | 2022 | Quantification of how mechanical ventilation influences the airborne infection risk of COVID-19 and HVAC energy consumption in office buildings |
| Perepi and others | 2022 | Predictive analysis of COVID 19 disease based on mathematical modelling and machine learning techniques |
| Pettit and others | 2021 | Optimized Post-Vaccination Strategies and Preventative Measures for SARS-CoV-2 |
| Plank and others | 2022 | Potential reduction in transmission of COVID-19 by digital contact tracing systems: a modelling study |
| Pung and others | 2022 | Using high-resolution contact networks to evaluate SARS-CoV-2 transmission and control in large-scale multi-day events |
| Quilty and others | 2021 | Quarantine and testing strategies to reduce transmission risk from imported SARS-CoV-2 infections: a global modelling study |
| Raymenants and others | 2022 | Empirical evidence on the efficiency of backward contact tracing in COVID-19 |
| Reyna-Lara and others | 2021 | Virus spread versus contact tracing: two competing contagion processes |
| Ridenti and others | 2022 | Mathematical Modeling and Investigation on the Role of Demography and Contact Patterns in Social Distancing Measures Effectiveness in COVID-19 Dissemination |
| Robinson and others | 2021 | Pilot evaluation of risk assessment and enhanced protocols regarding contacts at an international professional golf event |
| Rocha-Melogno and others | 2021 | Quantitative risk assessment of COVID-19 aerosol transmission indoors: a mechanistic stochastic web application |
| Seaman and others | 2023 | Decision Making across Adulthood during Physical Distancing |
| Shayak and others | 2022 | Contact Tracing Can Explain Counter-Intuitive COVID-19 Trajectories, Mitigate Disease Transmission and Provide an Early Warning Indicator - A Mathematical Modeling Study |
| Shiva and others | 2022 | The Luxury of Lockdown |
| Sobolik and others | 2021 | Controlling risk of SARS-CoV-2 infection in essential workers of enclosed food manufacturing facilities |
| Stabile and others | 2021 | Ventilation procedures to minimize the airborne transmission of viruses in classrooms |
| Supatgiat | 2021 | Effects of control measures and their impacts on COVID-19 transmission dynamics |
| Szczuka and others | 2023 | Handwashing adherence during the COVID-19 pandemic: A longitudinal study based on protection motivation theory |
| Tavori and others | 2021 | Super-Spreaders Out, Super-Spreading In: The Effects of Infectiousness Heterogeneity and Lockdowns on Herd Immunity |
| Torneri and others | 2022 | Controlling SARS-CoV-2 in schools using repetitive testing strategies |
| Vanni and others | 2021 | Human Mobility and Epidemic Evolution |
| Villers and others | 2022 | SARS-CoV-2 aerosol transmission in schools: the effectiveness of different interventions |
| Yasutaka and others | 2021 | Assessment of COVID-19 risk and prevention effectiveness among spectators of mass gathering events |
| Yeung and others | 2021 | Machine learning-based prediction of growth in confirmed COVID-19 infection cases in 114 countries using metrics of nonpharmaceutical interventions and cultural dimensions: Model development and validation |
| **Not COVID-19 n=13** | | |
| Ahmadzadeh and others | 2021 | Passenger exposure to respiratory aerosols in a train cabin: Effects of window, injection source, output flow location |
| Arsene and others | 2021 | Viral kinetic modeling and clinical trial simulation predicts disruption of respiratory disease trials by non-pharmaceutical COVID-19 interventions |
| Bandiera and others | 2020 | Face coverings and respiratory tract droplet dispersion |
| Beale and others | 2020 | Hand Hygiene Practices and the Risk of Human Coronavirus Infections in a UK Community Cohort |
| Best and others | 2021 | The impact of varying class sizes on epidemic spread in a university population |
| Coyle and others | 2021 | Reduction of exposure to simulated respiratory aerosols using ventilation, physical distancing, and universal masking |
| Edwards and others | 2021 | Reducing COVID-19 Airborne Transmission Risks on Public Transportation Buses: An Empirical Study on Aerosol Dispersion and Control |
| Fierce and others | 2021 | High efficacy of layered controls for reducing transmission of airborne pathogens |
| Kong and others | 2021 | Localized and Whole-Room Effects of Portable Air Filtration Units on Aerosol Particle Deposition and Concentration in a Classroom Environment |
| Lindsley and others | 2021 | Efficacy of universal masking for source control and personal protection from simulated cough and exhaled aerosols in a room |
| Motamedi and others | 2022 | CFD modeling of airborne pathogen transmission of COVID-19 in confined spaces under different ventilation strategies |
| Park and others | 2021 | Natural ventilation strategy and related issues to prevent coronavirus disease 2019 (COVID-19) airborne transmission in a school building |
| Sinha and others | 2021 | Mass testing and proactiveness affect epidemic spreading |
| **Health or social care settings n=6** | | |
| Ahmad and others | 2022 | Benefits of inpatient contact tracing and illustration of social inequalities and their relation to increasing risk of hospitalisation by COVID-19 |
| Alhakmi and others | 2022 | Exploring COVID-19 lateral flow testing engagement and compliance in selected Imperial College Healthcare Trust wards |
| Guo and others | 2021 | Using portable air purifiers to reduce airborne transmission of infectious respiratory viruses – a computational fluid dynamics study |
| Hellewell and others | 2021 | Estimating the effectiveness of routine asymptomatic PCR testing at different frequencies for the detection of SARS-CoV-2 infections |
| Higham and others | 2022 | Rapid qualitative analysis in a mixed-methods evaluation of an infection prevention intervention in a UK hospital setting during the COVID-19 pandemic: A discussion of the CLEAN study methodology |
| Micocci and others | 2021 | COVID-19 testing in English care homes and implications for staff and residents |
| **Wrong exposure n=155** | | |
| Adamson and others | 2022 | A large outbreak of COVID-19 in a UK prison, October 2020 to April 2021 |
| Aganovic and others | 2021 | Estimating the impact of indoor relative humidity on SARS-CoV-2 airborne transmission risk using a new modification of the Wells-Riley model |
| Agrawal and others | 2022 | SUTRA: A Novel Approach to Modelling Pandemics with Applications to COVID-19 |
| Ainsworth and others | 2021 | Infection control behavior at home during the COVID-19 pandemic: Observational study of a web-based behavioral intervention (Germ defence) |
| Akindeinde and others | 2021 | Fractional SEIRP model for COVID-19 dynamics incorporating social distancing and environment |
| Amaral and others | 2021 | An epidemiological model with voluntary quarantine strategies governed by evolutionary game dynamics |
| Anderson and others | 2021 | How much leeway is there to relax COVID-19 control measures? |
| Armitage and others | 2021 | Identifying targets for interventions to support public adherence to government instructions to reduce transmission of SARS-CoV-2 |
| Ashby and others | 2021 | Non-pharmaceutical interventions and the emergence of pathogen variants |
| Bajaj and others | 2021 | Context-specific emergence and growth of the SARS-CoV-2 Delta variant |
| Banks and others | 2022 | Modelling plausible scenarios for the Omicron SARS-CoV-2 variant from early-stage surveillance |
| Basellini and others | 2021 | Linking excess mortality to mobility data during the first wave of COVID-19 in England and Wales |
| Batteux and others | 2022 | Impact of residual risk messaging to reduce false reassurance following test-negative results from asymptomatic coronavirus (SARS-CoV-2) testing: An online experimental study of a hypothetical test |
| Bin and others | 2021 | Hysteresis-based supervisory control with application to non-pharmaceutical containment of COVID-19 |
| Birrell and others | 2021 | Real-time nowcasting and forecasting of COVID-19 dynamics in England: the first wave |
| Blomquist and others | 2021 | Risk of symptomatic COVID-19 due to aircraft transmission: a retrospective cohort study of contact-traced flights during England's containment phase |
| Bondaronek and others | 2022 | User feedback on the NHS Test & Trace Service during COVID-19: the use of machine learning to analyse free-text data from 37,914 UK adults |
| Brooks-Pollock and others | 2023 | Voluntary risk mitigation behaviour can reduce impact of SARS-CoV-2: a real-time modelling study of the January 2022 Omicron wave in England |
| Brown and others | 2021 | Inactivation of SARS-CoV-2 in chlorinated swimming pool water |
| Buchan and others | 2021 | Improved estimates of 222 nm far-UVC susceptibility for aerosolized human coronavirus via a validated high-fidelity coupled radiation-CFD code |
| Cano and others | 2020 | Covid-19 modelling: The effects of social distancing |
| Carvalho and others | 2021 | Analysis and Forecasting Incidence, Intensive Care Unit Admissions, and Projected Mortality Attributable to COVID-19 in Portugal, the UK, Germany, Italy, and France: Predictions for 4 Weeks Ahead |
| Chalkiadakis and others | 2021 | Infection rate models for COVID-19: Model risk and public health news sentiment exposure adjustments |
| Challen and others | 2021 | Estimates of regional infectivity of COVID-19 in the United Kingdom following imposition of social distancing measures |
| Chudik and others | 2021 | COVID-19 Time-varying Reproduction Numbers Worldwide: An Empirical Analysis of Mandatory and Voluntary Social Distancing |
| Coccia | 2022 | Restriction policies and effects of COVID-19 pandemic in environment: analysis and role of sustainable technology to cope with future pandemics |
| Collier and others | 2022 | Point of care SARS-CoV-2 nucleic acid testing in schools improves school attendance |
| Congdon | 2021 | Mid-Epidemic Forecasts of COVID-19 Cases and Deaths: A Bivariate Model Applied to the UK |
| Conroy and others | 2022 | Very small effects of an imagery-based randomised trial to promote adherence to wearing face coverings during the COVID-19 pandemic and identification of future intervention targets |
| Cordery and others | 2022 | Transmission of SARS-CoV-2 by children to contacts in schools and households: a prospective cohort and environmental sampling study in London |
| Davies and others | 2023 | Observed and self-reported COVID-19 health protection behaviours on a university campus and the impact of a single simple intervention |
| Demis and others | 2021 | A semi-continuous model for transmission of SARS-CoV-2 and other respiratory viruses in enclosed spaces via multiple pathways to assess risk of infection and mitigation strategies |
| Dimarco and others | 2021 | Optimal control of epidemic spreading in presence of social heterogeneity |
| Ding and others | 2021 | Factors affecting adherence to non-pharmaceutical interventions for COVID-19 infections in the first year of the pandemic in the UK |
| Docquier and others | 2021 | Are Travel Bans the Answer to Stopping the Spread of COVID-19 Variants? Lessons from a Multi-Country SIR Model |
| Donnat and others | 2021 | Modeling the Heterogeneity in COVID-19's Reproductive Number and its Impact on Predictive Scenarios |
| Drakesmith and others | 2021 | Developing a population data science approach to assess increased risk of COVID-19 associated with attending large events |
| Drews and others | 2022 | Model-based ensembles: Lessons learned from retrospective analysis of COVID-19 infection forecasts across 10 countries |
| du and others | 2021 | Establishment and lineage dynamics of the SARS-CoV-2 epidemic in the UK |
| Duran-Olivencia and others | 2021 | Understanding Soaring Coronavirus Cases and the Effect of Contagion Policies in the UK |
| Dutta and others | 2021 | Using mobility data in the design of optimal lockdown strategies for the COVID-19 pandemic |
| Dye and others | 2020 | The scale and dynamics of COVID-19 epidemics across Europe |
| Edmunds and others | 2021 | The COVID University Challenge: A Hazard Analysis of Critical Control Points Assessment of the Return of Students to Higher Education Establishments |
| Ellis and others | 2021 | The course of the UK COVID 19 pandemic; no measurable impact of new variants |
| Fair and others | 2021 | Population behavioural dynamics can mediate the persistence of emerging infectious diseases |
| Febres | 2021 | Assessing the impact of social activity permissiveness on the COVID-19 infection curve of several countries |
| Feng and others | 2021 | Removal of SARS-CoV-2 using UV+Filter in built environment |
| Fierce and others | 2021 | Simulating near-field enhancement in transmission of airborne viruses with a quadrature-based model |
| Fisher and others | 2021 | Experiences of the coronavirus disease-19 (COVID-19) pandemic from the perspectives of young people: Rapid qualitative study |
| Foat and others | 2022 | Modeling the effect of temperature and relative humidity on exposure to SARS-CoV-2 in a mechanically ventilated room |
| Fokas and others | 2021 | Covid-19: predictive mathematical formulae for the number of deaths during lockdown and possible scenarios for the post-lockdown period |
| Freeman and others | 2021 | Communicating personalised risks from COVID-19: guidelines from an empirical study |
| Fujii and others | 2021 | Public perceptions, individual characteristics, and preventive behaviors for COVID-19 in six countries: a cross-sectional study |
| Gallic and others | 2021 | Optimal lockdowns for COVID-19 pandemics: Analyzing the efficiency of sanitary policies in Europe |
| Gartland and others | 2023 | Experiences, Perceptions of Risk, and Lasting Impacts of COVID-19 for Employees in the Public Transport Sector |
| Gerli and others | 2021 | Forecasting COVID-19 infection trends and new hospital admissions in England due to SARS-CoV-2 Variant of Concern Omicron |
| Gerli and others | 2021 | Forecasting COVID-19 infection trends in the EU-27 countries, the UK and Switzerland due to SARS-CoV-2 Variant of Concern Omicron |
| Gomes and others | 2022 | Individual variation in susceptibility or exposure to SARS-CoV-2 lowers the herd immunity threshold |
| Griette and others | 2021 | What can we learn from COVID-19 data by using epidemic models with unidentified infectious cases? |
| Griffith and others | 2022 | Continuing inequalities in COVID-19 mortality in England and Wales, and the changing importance of regional, over local, deprivation |
| Guerstein and others | 2021 | The interplay between vaccination and social distancing strategies affects COVID19 population-level outcomes |
| Guimaraes | 2021 | Antibody tests: They are more important than we thought |
| Habib and others | 2021 | Non-linear spatial linkage between COVID-19 pandemic and mobility in ten countries: A lesson for future wave |
| Hansen and others | 2021 | Nudging hand hygiene compliance: a large-scale field experiment on hospital visitors |
| Haroon and others | 2020 | Estimating The Possible Role Of Testing Capacity And Social Distancing In Predicting The Growth Rate Of Daily Covid-19 Cases |
| Howerton and others | 2021 | Synergistic interventions to control COVID-19: Mass testing and isolation mitigates reliance on distancing |
| Hubert and others | 2022 | The effects of organizational climate on adherence to guidelines for COVID-19 prevention |
| Huberts and others | 2023 | Optimal timing of non-pharmaceutical interventions during an epidemic |
| Iddon and others | 2022 | A population framework for predicting the proportion of people infected by the far-field airborne transmission of SARS-CoV-2 indoors |
| Issakhov and others | 2022 | Assessment of airborne transmission from coughing processes with thermal plume adjacent to body and radiators on effectiveness of social distancing |
| Jones and others | 2021 | Modelling uncertainty in the relative risk of exposure to the SARS-CoV-2 virus by airborne aerosol transmission in well mixed indoor air |
| Keeling and others | 2020 | Efficacy of contact tracing for the containment of the 2019 novel coronavirus (COVID-19) |
| Keeling and others | 2021 | Predictions of COVID-19 dynamics in the UK: Short-term forecasting and analysis of potential exit strategies |
| Keeling and others | 2022 | Comparison of the 2021 COVID-19 roadmap projections against public health data in England |
| Kerr and others | 2022 | Common protocol for validation of the QCOVID algorithm across the four UK nations |
| Khataee and others | 2021 | Effects of social distancing on the spreading of COVID-19 inferred from mobile phone data |
| Kraemer and others | 2021 | Spatiotemporal invasion dynamics of SARS-CoV-2 lineage B.1.1.7 emergence |
| Laha and others | 2023 | A multi-type branching process model for epidemics with application to COVID-19 |
| Laroze and others | 2021 | COVID-19 does not stop at open borders: Spatial contagion among local authority districts during England's first wave |
| Lecouturier and others | 2021 | Public understanding of COVID-19 antibody testing and test results: A qualitative study conducted in the U.K. early in the pandemic |
| Lee and others | 2021 | Computational modelling of COVID-19: A study of compliance and superspreaders |
| Lee and others | 2022 | Severe Acute Respiratory Syndrome Coronavirus 2 (SARS-CoV-2) Infectivity by Viral Load, S Gene Variants and Demographic Factors, and the Utility of Lateral Flow Devices to Prevent Transmission |
| Leeman and others | 2022 | Effect of Returning University Students on COVID-19 Infections in England, 2020 |
| Lewandowsky and others | 2021 | Public acceptance of privacy-encroaching policies to address the COVID-19 pandemic in the United Kingdom |
| Li and others | 2021 | The association of community mobility with the time-varying reproduction number (R) of SARS-CoV-2: a modelling study across 330 local UK authorities |
| Liu and others | 2021 | Transmission dynamics of the COVID-19 epidemic in England |
| Lopez and others | 2021 | Impact of isolating COVID-19 patients in a supervised community facility on transmission reduction among household members |
| Madden and others | 2022 | Smart Hand Sanitisers in the Workplace: A Survey of Attitudes towards an Internet of Things Technology |
| Manca and others | 2023 | Impact of perceptions and attitudes on air travel choices in the post-COVID-19 era: A cross-national analysis of stated preference data |
| Mansab and others | 2021 | Performance of national COVID-19 symptom checkers': A comparative case simulation study |
| Margraf and others | 2020 | Behavioral measures to fight COVID-19: An 8-country study of perceived usefulness, adherence and their predictors |
| Margraf and others | 2021 | Adherence to behavioral Covid-19 mitigation measures strongly predicts mortality |
| Mazzoli and others | 2021 | Interplay between mobility, multi-seeding and lockdowns shapes COVID-19 local impact |
| Mc Goldrick and others | 2022 | Surveillance of COVID-19 cases associated with dental settings using routine health data from the East of Scotland with a description of efforts to break chains of transmission from October 2020 to December 2021 |
| McAleavey and others | 2022 | Outbreak of SARS-CoV-2 in a teenage discotheque in Northern Ireland-November 2021 |
| Miles and others | 2021 | Assessing the spread of the novel coronavirus in the absence of mass testing |
| Mishra and others | 2021 | Comparing the responses of the UK, Sweden and Denmark to COVID-19 using counterfactual modelling |
| Moore and others | 2021 | Vaccination and non-pharmaceutical interventions for COVID-19: a mathematical modelling study |
| Morgan and others | 2021 | Optimizing time-limited non-pharmaceutical interventions for COVID-19 outbreak control |
| Morrissey and others | 2021 | Area level deprivation and monthly COVID-19 cases: The impact of government policy in England |
| Morton and others | 2021 | Infection control in the home: A qualitative study exploring perceptions and experiences of adhering to protective behaviours in the home during the COVID-19 pandemic |
| Mowbray and others | 2021 | Is My Cough a Cold or Covid? A Qualitative Study of COVID-19 Symptom Recognition and Attitudes Toward Testing in the UK |
| Muis and others | 2022 | Flattening the COVID-19 curve: Emotions mediate the effects of a persuasive message on preventive action |
| Nashebi and others | 2022 | Using a real-world network to model the tradeoff between stay-at-home restriction, vaccination, social distancing and working hours on COVID-19 dynamics |
| Nightingale and others | 2021 | The importance of saturating density dependence for population-level predictions of SARS-CoV-2 resurgence compared with density-independent or linearly density-dependent models, England, 23 March to 31 July 2020 |
| Niu and others | 2022 | Ranking the effectiveness of non-pharmaceutical interventions to counter COVID-19 in UK universities with vaccinated population |
| Pellis and others | 2021 | Challenges in control of COVID-19: short doubling time and long delay to effect of interventions |
| Pesaran and others | 2022 | Matching theory and evidence on Covid-19 using a stochastic network SIR model |
| Picchiotti and others | 2021 | COVID-19 pandemic: a mobility-dependent SEIR model with undetected cases in Italy, Europe and US |
| Raja and others | 2022 | Investigation of a SARS-CoV-2 Outbreak at an Automotive Manufacturing Site in England |
| Recchia and others | 2021 | How do the UK public interpret COVID-19 test results? Comparing the impact of official information about results and reliability used in the UK, USA and New Zealand: A randomised controlled trial |
| Ricks and others | 2021 | Quantifying the potential value of antigen-detection rapid diagnostic tests for COVID-19: a modelling analysis |
| Riley and others | 2021 | REACT-1 round 9 interim report: downward trend of SARS-CoV-2 in England in February 2021 but still at high prevalence |
| Riley and others | 2021 | REACT-1 round 8 interim report: SARS-CoV-2 prevalence during the initial stages of the third national lockdown in England |
| Rinaldi and others | 2022 | Epidemiological model based periodic intervention policies for COVID-19 mitigation in the United Kingdom |
| Rose and others | 2021 | Analysing COVID-19 outcomes in the context of the 2019 Global Health Security (GHS) Index |
| Ross and others | 2021 | Household visitation during the COVID-19 pandemic |
| Rossberg and others | 2020 | How will this continue? Modelling interactions between the COVID-19 pandemic and policy responses |
| Routledge and others | 2021 | Management of a large outbreak of COVID-19 at a British Army training centre: lessons for the future |
| Ruktanonchai and others | 2020 | Assessing the impact of coordinated COVID-19 exit strategies across Europe |
| Sachak-Patwa and others | 2021 | The risk of SARS-CoV-2 outbreaks in low prevalence settings following the removal of travel restrictions |
| Samartsidis and others | 2021 | Evaluating the impact of local tracing partnerships on the performance of contact tracing for COVID-19 in England |
| Sartorius and others | 2021 | Modelling and predicting the spatio-temporal spread of COVID-19, associated deaths and impact of key risk factors in England |
| Schmidtke and others | 2021 | A cross-sectional survey assessing the influence of theoretically informed behavioural factors on hand hygiene across seven countries during the COVID-19 pandemic |
| Secco and others | 2022 | To lockdown or not to lockdown: Analysis of the EU lockdown performance vs. COVID-19 outbreak |
| Sereno and others | 2021 | Model predictive control for optimal social distancing in a type SIR-switched model |
| Silk and others | 2022 | Observations and conversations: how communities learn about infection risk can impact the success of non-pharmaceutical interventions against epidemics |
| Sk and others | 2022 | The impact of a power law-induced memory effect on the SARS-CoV-2 transmission |
| Sloof and others | 2022 | Impact of supplementary air filtration on airborne particulate matter in a UK hospital ward |
| Smith and others | 2020 | Factors associated with adherence to self-isolation and lockdown measures in the UK: a cross-sectional survey |
| Smith and others | 2022 | Public health impact of mass sporting and cultural events in a rising COVID-19 prevalence in England |
| Spiliotis and others | 2021 | Optimal vaccine roll-out strategies with respect to social distancing measures for SARS-CoV-2 pandemic |
| Taylor and others | 2021 | Cross sectional investigation of a COVID-19 outbreak at a London Army barracks: Neutralising antibodies and virus isolation |
| Taylor and others | 2022 | Retrospective spatial analysis of cases of COVID-19 in a single military accommodation block corridor, RMAS, January-March 21 |
| Taylor and others | 2023 | A SARS-CoV-2 outbreak associated with five air force bases and a nightclub following the lifting of COVID-19-related social restrictions, United Kingdom, July-to-September 2021 |
| Thom and others | 2021 | Exploratory comparison of Healthcare costs and benefits of the UK's Covid-19 response with four European countries |
| Treneman-Evans and others | 2022 | The Rapid Adaptation and Optimisation of a Digital Behaviour-Change Intervention to Reduce the Spread of COVID-19 in Schools |
| Vandrevala and others | 2022 | Willingness of the UK public to volunteer for testing in relation to the COVID-19 pandemic |
| Venigalla and others | 2021 | SurviveCovid-19 -- An Educational Game to Facilitate Habituation of Social Distancing and Other Health Measures for Covid-19 Pandemic |
| Walker and others | 2021 | Remote data collection during COVID-19 restrictions: an example from a refugee and asylum-seeker participant group in the UK |
| Wang and others | 2021 | Heterogeneous interventions reduce the spread of COVID-19 in simulations on real mobility data |
| Ward and others | 2021 | Growth, reproduction numbers and factors affecting the spread of SARSCoV-2 novel variants of concern in the UK from October 2020 to July 2021: A modelling analysis |
| Weber | 2021 | Assessing the lockdown effect from excess mortalities |
| Welsh and others | 2021 | The effects of the first national lockdown in England on geographical inequalities in the evolution of COVID-19 case rates: An ecological study |
| Wey and others | 2021 | The benefits of peer transparency in safe workplace operation post pandemic lockdown |
| Whittaker and others | 2023 | Uncertainty and error in SARS-CoV-2 epidemiological parameters inferred from population-level epidemic models |
| Wilburn and others | 2021 | COVID-19 within a large UK prison with a high number of vulnerable adults, march to june 2020: An outbreak investigation and screening event |
| Williams and others | 2020 | Public perceptions and experiences of social distancing and social isolation during the COVID-19 pandemic: a UK-based focus group study |
| Wood and others | 2022 | Turn Up the Lights, Leave them On and Shine them All Around-Numerical Simulations Point the Way to more Efficient Use of Far-UVC Lights for the Inactivation of Airborne Coronavirus |
| Wright and others | 2020 | What predicts adherence to COVID-19 government guidelines? Longitudinal analyses of 51,000 UK adults |
| Yang and others | 2021 | The effect of multiple interventions to balance healthcare demand for controlling COVID-19 outbreaks: a modelling study |
| Yang and others | 2022 | Critical policies disparity of the first and second waves of COVID-19 in the United Kingdom |
| Young and others | 2021 | Interplay between COVID-19 vaccines and social measures for ending the SARS-CoV-2 pandemic |
| Younie and others | 2020 | Improving young children's handwashing behaviour and understanding of germs: The impact of A Germ's Journey educational resources in schools and public spaces |
| Zhao and others | 2022 | The global transmission of new coronavirus variants |
| **Wrong outcomes n=95** | | |
| Adenaiye and others | 2021 | Infectious SARS-CoV-2 in Exhaled Aerosols and Efficacy of Masks During Early Mild Infection |
| Adzic and others | 2022 | A post-occupancy study of ventilation effectiveness from high-resolution CO_2_ monitoring at live theatre events to mitigate airborne transmission of SARS-CoV-2 |
| Aiano and others | 2021 | Feasibility and acceptability of SARS-CoV-2 testing and surveillance in primary school children in England: Prospective, crosssectional study |
| Amin-Chowdhury and others | 2022 | Parents' and teachers' attitudes to and experiences of the implementation of COVID-19 preventive measures in primary and secondary schools following reopening of schools in autumn 2020: a descriptive cross-sectional survey |
| Atchison and others | 2023 | Validity of Self-testing at Home With Rapid Severe Acute Respiratory Syndrome Coronavirus 2 Antibody Detection by Lateral Flow Immunoassay |
| Bachtiger and others | 2020 | Belief of having had unconfirmed Covid-19 infection reduces willingness to participate in app-based contact tracing |
| Berg-Beckhoff and others | 2022 | Political stringency, infection rates, and higher education students' adherence to government measures in the Nordic countries and the UK during the first wave of the COVID-19 outbreak |
| Berry and others | 2022 | Facilitators and barriers to social distancing for young people during the COVID-19 pandemic |
| Bowman and others | 2021 | Public perceptions and preventive behaviours during the early phase of the COVID-19 pandemic: a comparative study between Hong Kong and the United Kingdom |
| Brooks-Pollock and others | 2021 | The population attributable fraction of cases due to gatherings and groups with relevance to COVID-19 mitigation strategies |
| Buhler and others | 2021 | Stay Out of the Blast Radius: Influence of Surgical Masks on Virtual Pedestrian Interactions |
| Burridge and others | 2022 | Public efforts to reduce disease transmission implied from a spatial game |
| Bushnaq and others | 2021 | Control of COVID-19 dynamics through a fractional-order model |
| Carter and others | 2021 | Experiences of supported isolation in returning travellers during the early COVID-19 response: A qualitative interview study |
| Cheng and others | 2022 | Human mobility variations in response to restriction policies during the COVID-19 pandemic: An analysis from the Virus Watch community cohort in England, UK |
| Cox and others | 2022 | Mixed-methods exploration of views on choice in a university asymptomatic COVID-19 testing programme |
| Davies and others | 2021 | Acceptability, Usability, and Performance of Lateral Flow Immunoassay Tests for Severe Acute Respiratory Syndrome Coronavirus 2 Antibodies: REACT-2 Study of Self-Testing in Nonhealthcare Key Workers |
| den Daas and others | 2022 | An experimental COVID-19 messaging study in a representative sample of the Scottish population: Increasing physical distancing intentions through self-efficacy |
| Dennis and others | 2022 | The social media response to twice-weekly mass asymptomatic testing in England |
| Dowthwaite and others | 2021 | Public adoption of and trust in the nhs covid-19 contact tracing app in the united kingdom: Quantitative online survey study |
| Dowthwaite and others | 2022 | The relationship between trust and attitudes towards the COVID-19 digital contact-tracing app in the UK |
| Eales and others | 2022 | Trends in SARS-CoV-2 infection prevalence during England's roadmap out of lockdown, January to July 2021 |
| Edelman and others | 2021 | Network analysis of England’s single parent household COVID-19 control policy impact: a proof-of-concept study |
| Egan and others | 2021 | Evaluating the effect of infographics on public recall, sentiment and willingness to use face masks during the COVID-19 pandemic: a randomised internet-based questionnaire study |
| Emerson and others | 2021 | The impact of disability on employment and financial security following the outbreak of the 2020 COVID-19 pandemic in the UK |
| Eshareturi and others | 2021 | An exploration of the impact of SARS-CoV-2 (COVID-19) restrictions on marginalised groups in the UK |
| Essa and others | 2021 | What is the effect of lockdown upon hospitalisation because of COVID-19 amongst patients from a heart failure registry? |
| Fenton and others | 2021 | A Bayesian network model for personalised COVID19 risk assessment and contact tracing |
| Ferguson and others | 2021 | Validation testing to determine the sensitivity of lateral flow testing for asymptomatic SARSCoV-2 detection in low prevalence settings: Testing frequency and public health messaging is key |
| Filho and others | 2021 | A Transnational and Transregional Study of the Impact and Effectiveness of Social Distancing for COVID-19 Mitigation |
| Foad and others | 2021 | The limitations of polling data in understanding public support for COVID-19 lockdown policies |
| Green and others | 2021 | Evaluating social and spatial inequalities of large scale rapid lateral flow SARS-CoV-2 antigen testing in COVID-19 management: An observational study of Liverpool, UK (November 2020 to January 2021) |
| Green and others | 2022 | Trends in inequalities in avoidable hospitalisations across the COVID-19 pandemic: A cohort study of 23.5 million people in England |
| Hajdu and others | 2022 | Contextual factors predicting compliance behavior during the COVID-19 pandemic: A machine learning analysis on survey data from 16 countries |
| Halford and others | 2022 | Understanding reported COVID-19 cases in England following changes to testing, between November 2021 and April 2022 |
| Harris | 2021 | Experiences with testing, self-isolation and vaccination in north east england during the covid pandemic |
| Haw and others | 2021 | The costs of keeping schools open during the COVID-19 pandemic |
| Henriques and others | 2022 | Modelling airborne transmission of SARS-CoV-2 using CARA: risk assessment for enclosed spaces |
| Ho and others | 2021 | Spatiotemporal droplet dispersion measurements demonstrate face masks reduce risks from singing: results from the COvid aNd FacEmaSkS Study (CONFESS) |
| Horvath and others | 2022 | Adoption and continued use of mobile contact tracing technology: Multilevel explanations from a three-wave panel survey and linked data |
| Howard | 2022 | The relations between age, face mask perceptions and face mask wearing |
| Iqbal and others | 2021 | The pilot, proof of concept REMOTE-COVID trial: remote monitoring use in suspected cases of COVID-19 (SARS-CoV 2) |
| Isherwood and others | 2021 | Challenges to self-isolation among contacts of cases of COVID-19: a national telephone survey in Wales |
| Jaspal and others | 2022 | Social support, perceived risk and the likelihood of COVID-19 testing and vaccination: cross-sectional data from the United Kingdom |
| Jayes and others | 2022 | Perspectives of attenders and non-attenders to SARS-CoV-2 asymptomatic community testing in England: a qualitative interview study |
| Jones and others | 2021 | Durham University students' experiences of asymptomatic COVID-19 testing: A qualitative study |
| Jones and others | 2021 | To Use or Not to Use a COVID-19 Contact Tracing App: Mixed Methods Survey in Wales |
| Juul and others | 2022 | Comparing the efficiency of forward and backward contact tracing |
| Kale and others | 2022 | Patterns and predictors of adherence to health-protective measures during COVID-19 pandemic in the UK: cross-sectional and longitudinal findings from the HEBECO study |
| Keyworth and others | 2021 | What challenges do UK adults face when adhering to COVID-19-related instructions? Cross-sectional survey in a representative sample |
| Kim and others | 2021 | Automated Contact Tracing: a game of big numbers in the time of COVID-19 |
| Ladhani and others | 2021 | SARS-CoV-2 infection and transmission in primary schools in England in June-December, 2020 (sKIDs): an active, prospective surveillance study |
| Lasseter and others | 2022 | Exploring the impact of shielding advice on the wellbeing of individuals identified as clinically extremely vulnerable amid the COVID-19 pandemic: a mixed-methods evaluation |
| Lawson and others | 2021 | An evaluation of the hand hygiene behaviour and compliance of the general public when using public restrooms in Northern Ireland (NI) during the initial weeks of the novel coronavirus (covid-19) pandemic |
| Lee and others | 2021 | The association between socioeconomic status and mobility reductions in the early stage of England's COVID-19 epidemic |
| Lin and others | 2021 | Public Attitudes and Factors of COVID-19 Testing Hesitancy in the United Kingdom and China: Comparative Infodemiology Study |
| Lloyd and others | 2021 | Delay discounting and under-valuing of recent information predict poorer adherence to social distancing measures during the COVID-19 pandemic |
| Mancastroppa and others | 2021 | Stochastic sampling effects favor manual over digital contact tracing |
| Marchant and others | 2021 | Primary school staff perspectives of school closures due to COVID-19, experiences of schools reopening and recommendations for the future: A qualitative survey in Wales |
| Megna | 2021 | Inferring a cause-effect relationship between lockdown restrictions and COVID-19 pandemic trend during the first wave |
| Miles and others | 2021 | "Stay at Home, Protect the National Health Service, Save Lives": A cost benefit analysis of the lockdown in the United Kingdom |
| Moshe and others | 2021 | SARS-CoV-2 lateral flow assays for possible use in national covid-19 seroprevalence surveys (React 2): Diagnostic accuracy study |
| Osterrieder and others | 2021 | Economic and social impacts of COVID-19 and public health measures: results from an anonymous online survey in Thailand, Malaysia, the UK, Italy and Slovenia |
| Palmateer and others | 2021 | National population prevalence of antibodies to SARS-CoV-2 in Scotland during the first and second waves of the COVID-19 pandemic |
| Pepper and others | 2022 | Understanding Trust and Changes in Use After a Year With the NHS COVID-19 Contact Tracing App in the United Kingdom: Longitudinal Mixed Methods Study |
| Perez-Guzman and others | 2023 | Epidemiological drivers of transmissibility and severity of SARS-CoV-2 in England |
| Persing and others | 2022 | Evaluation of ventilation, indoor air quality, and probability of viral infection in an outdoor dining enclosure |
| Peto and others | 2021 | COVID-19: Rapid antigen detection for SARS-CoV-2 by lateral flow assay: A national systematic evaluation of sensitivity and specificity for mass-testing |
| Powell and others | 2022 | Secondary attack rates in primary and secondary school bubbles following a confirmed case: Active, prospective national surveillance, November to December 2020, England |
| Prosser and others | 2023 | Estimating the risk reduction of isolation on COVID-19 nonhousehold transmission and severe/critical illness in nonimmune individuals: September to November 2021 |
| Purves and others | 2023 | Attending sporting mega events during COVID-19: mitigation and messaging at UK EURO 2020 matches |
| Rencken and others | 2021 | Patterns of SARS-CoV-2 Aerosol Spread in Typical Classrooms |
| Robin and others | 2022 | Understanding adherence to self-isolation in the first phase of COVID-19 response |
| Robinson and others | 2022 | How effective are face coverings in reducing transmission of COVID-19? |
| Rusu and others | 2021 | Modelling digital and manual contact tracing for COVID-19. Are low uptakes and missed contacts deal-breakers? |
| Rutter and others | 2022 | Using FaceReader to explore the potential for harnessing emotional reactions to motivate hand hygiene |
| Scarabel and others | 2021 | A renewal equation model to assess roles and limitations of contact tracing for disease outbreak control |
| Schneiders and others | 2022 | The impact of COVID-19 non-pharmaceutical interventions on the lived experiences of people living in Thailand, Malaysia, Italy and the United Kingdom: A cross-country qualitative study. |
| Senior | 2023 | Self-completed online contact tracing for COVID-19 is associated with reporting fewer contacts: an observational study |
| Smith and others | 2022 | Tiered restrictions for COVID-19 in England: knowledge, motivation and self-reported behaviour |
| Smith and others | 2022 | Intention to adhere to test, trace, and isolate during the COVID-19 pandemic (the COVID-19 Rapid Survey of Adherence to Interventions and Responses study) |
| Smith and others | 2022 | Mask communication: The development of the face covering as a semiotic resource through government public health posters in England and Wales |
| Sturniolo and others | 2021 | Testing, tracing and isolation in compartmental models |
| Sundaram and others | 2021 | Implementation of preventive measures to prevent COVID-19: a national study of English primary schools in summer 2020 |
| Sweeney and others | 2021 | Exploring equity in health and poverty impacts of control measures for SARS-CoV-2 in six countries |
| Taamouti | 2021 | Covid-19 Control and the Economy: Test, Test, Test |
| Thomas and others | 2021 | Social, demographic and behavioural determinants of SARS-CoV-2 infection: A case-control study carried out during mass community testing of asymptomatic individuals in South Wales, December 2020 |
| Thorneloe and others | 2022 | Adherence to behaviours associated with the test, trace, and isolate system: an analysis using the theoretical domains framework |
| Tildesley and others | 2022 | Optimal health and economic impact of non-pharmaceutical intervention measures prior and post vaccination in England: a mathematical modelling study |
| Tupper and others | 2020 | Event-specific interventions to minimize COVID-19 transmission |
| Welsh and others | 2022 | Inequalities in the evolution of the COVID-19 pandemic: an ecological study of inequalities in mortality in the first wave and the effects of the first national lockdown in England |
| Williams and others | 2021 | Public perceptions of non-adherence to pandemic protection measures by self and others: A study of COVID-19 in the United Kingdom |
| Woodland and others | 2022 | What influences whether parents recognise COVID-19 symptoms, request a test and self-isolate: A qualitative study |
| Wright and others | 2021 | Do predictors of adherence to pandemic guidelines change over time? A panel study of 22,000 UK adults during the COVID-19 pandemic |
| Zarif and others | 2021 | The impact of primary care supported shielding on the risk of mortality in people vulnerable to COVID-19: English sentinel network matched cohort study |
| **Wrong study design n=107** | | |
| Abdeen and others | 2021 | An approximate analytical formula for estimating the weight of factors affecting the spread of COVID-19: a case study of the first wave |
| Agius and others | 2021 | Protection from covid-19 at work: Health and safety law is fit for purpose |
| Alfano | 2022 | The Effects of School Closures on COVID-19: A Cross-Country Panel Analysis |
| An and others | 2021 | Effects of Early Mask Mandates and Other Policy Interventions on COVID-19 Infections |
| Anjum and others | 2022 | IoT-Based COVID-19 Diagnosing and Monitoring Systems: A Survey |
| Anonymous | 2021 | Covid-19: How the UK is using lateral flow tests in the pandemic |
| Anonymous | 2022 | Correction: Who is engaging with lateral flow testing for COVID-19 in The UK? The COVID-19 Rapid Survey of Adherence to Interventions and Responses (CORSAIR) study |
| Aravindakshan and others | 2021 | The Impact of Mask-Wearing in Mitigating the Spread of COVID-19 During the Early Phases of the Pandemic |
| Bandyopadhyay | 2021 | Institutional racism and national lockdowns - Author's reply |
| Banerjee and others | 2022 | Data driven covid-19 spread prediction based on mobility and mask mandate information |
| Banholzer and others | 2021 | Estimating the effects of non-pharmaceutical interventions on the number of new infections with COVID-19 during the first epidemic wave |
| Barros and others | 2022 | A causal inference approach for estimating effects of non-pharmaceutical interventions during Covid-19 pandemic |
| Bendavid and others | 2021 | Assessing mandatory stay-at-home and business closure effects on the spread of COVID-19 |
| Bianconi and others | 2020 | Efficiency of COVID-19 mobile contact tracing containment by measuring time-dependent doubling time |
| Bikbov and others | 2021 | Maximum incubation period for COVID-19 infection: Do we need to rethink the 14-day quarantine policy? |
| Boesch | 2021 | Lockdown benefit varies among countries and sub-national units: a reanalysis of the data by Bendavid et al. (2021) |
| Brauner and others | 2021 | Inferring the effectiveness of government interventions against COVID-19 |
| Briggs and others | 2021 | Is NHS Test and Trace exacerbating COVID-19 inequalities? |
| Buonsenso and others | 2021 | Schools closures during the COVID-19 pandemic |
| Burg and others | 2023 | Trajectories of COVID-19: a longitudinal analysis of many nations and subnational regions |
| Burns and others | 2021 | Border control and SARS-CoV-2: An opportunity for generating highly policy-relevant, real-world evidence |
| Chattopadhyay and others | 2021 | Infection kinetics of Covid-19 and containment strategy |
| Chen and others | 2021 | Exploring the Drivers and Barriers to Uptake for Digital Contact Tracing |
| Chung and others | 2021 | Impact of physical distancing policy on reducing transmission of SARS-CoV-2 globally: Perspective from government's response and residents' compliance |
| Chung and others | 2021 | Effects of government policies on the spread of COVID-19 worldwide |
| Conway and others | 2021 | Epidemiology of COVID-19 and public health restrictions during the first wave of the pandemic in Ireland in 2020 |
| Costa and others | 2022 | Impact of non-pharmaceutical interventions on COVID-19 incidence and deaths: cross-national natural experiment in 32 European countries |
| De Leo | 2021 | Impact of COVID-19 Testing Strategies and Lockdowns on Disease Management Across Europe, South America, and the United States: Analysis Using Skew-Normal Distributions |
| Deeks and others | 2022 | SARS-CoV-2 antigen lateral flow tests for detecting infectious people: linked data analysis |
| Dickens and others | 2020 | Strategies at points of entry to reduce importation risk of COVID-19 cases and reopen travel |
| Drury and others | 2021 | Re-opening live events and large venues after Covid-19 'lockdown': Behavioural risks and their mitigations |
| Dyson | 2022 | Modelling results on the impact of COVID-19 testing in schools |
| Fearon and others | 2021 | SARS-CoV-2 antigen testing: weighing the false positives against the costs of failing to control transmission |
| Fedele and others | 2022 | COVID-19 NHS infection control strategy: Errare humanum est, perseverare autem diabolicum |
| Feeney and others | 2022 | Self-testing for covid-19: Adding oropharyngeal to nasal sampling is not the answer to underperforming tests |
| Flaxman and others | 2020 | Estimating the effects of non-pharmaceutical interventions on COVID-19 in Europe |
| Fountoulakis and others | 2020 | Factors determining different death rates because of the COVID-19 outbreak among countries |
| Galanti and others | 2021 | Social distancing remains key during vaccinations |
| Ge and others | 2021 | Untangling the changing impact of non-pharmaceutical interventions and vaccination on European Covid-19 trajectories |
| Ghosh and others | 2021 | Global-scale analysis and longitudinal assessment of COVID-19 incidence in the first six months |
| Harris and others | 2021 | Safe management of full-capacity live/mass events in COVID-19 will require mathematical, epidemiological and economic modelling |
| Hassan and others | 2021 | Efficacy the of Confinement Policies on the COVID-19 Spread Dynamics in the Early Period of the Pandemic |
| Hunter and others | 2021 | Impact of non-pharmaceutical interventions against COVID-19 in Europe in 2020: a quasi-experimental non-equivalent group and time series design study |
| Ibrahim and others | 2021 | Variational-LSTM autoencoder to forecast the spread of coronavirus across the globe |
| Ilyin | 2021 | A Recursive Model of the Spread of COVID-19: Modelling Study |
| Islam and others | 2020 | Physical distancing interventions and incidence of coronavirus disease 2019: natural experiment in 149 countries |
| Jain and others | 2022 | The global response: How cities and provinces around the globe tackled Covid-19 outbreaks in 2021 |
| Jamison and others | 2021 | Comparing the impact on COVID-19 mortality of self-imposed behavior change and of government regulations across 13 countries |
| Jayasinghe and others | 2021 | Bio-Politics and Calculative Technologies in COVID-19 Governance: Reflections From England |
| Kannoth and others | 2021 | The Association between Early Country-level Testing Capacity and Later COVID-19 Mortality Outcomes |
| Koh and others | 2020 | Estimating the impact of physical distancing measures in containing COVID-19: an empirical analysis |
| Kohanovski and others | 2022 | Inferring the effective start dates of non-pharmaceutical interventions during COVID-19outbreaks |
| Kumar and others | 2021 | Infection vulnerability stratification risk modelling of COVID-19 data: a deterministic SEIR epidemic model analysis |
| Lai and others | 2021 | Assessing the Effect of Global Travel and Contact Restrictions on Mitigating the COVID-19 Pandemic |
| Leech and others | 2022 | Mask wearing in community settings reduces SARS-CoV-2 transmission |
| Li and others | 2021 | The temporal association of introducing and lifting non-pharmaceutical interventions with the time-varying reproduction number (R) of SARS-CoV-2: a modelling study across 131 countries. |
| Li and others | 2021 | Forecasting COVID-19 and Analyzing the Effect of Government Interventions |
| Liang and others | 2021 | COVID-19 case doubling time associated with non-pharmaceutical interventions and vaccination: A global experience. |
| Liu and others | 2021 | The impact of non-pharmaceutical interventions on SARS-CoV-2 transmission across 130 countries and territories |
| Mader and others | 2022 | The Effects of Non-pharmaceutical Interventions on COVID-19 Mortality: A Generalized Synthetic Control Approach Across 169 Countries. |
| Majeed | 2022 | It's time for more targeted use of lateral flow tests for covid-19 |
| Martin and others | 2021 | Appropriate Usage of Face Masks to Prevent SARS-CoV-2: Sharpening the Messaging Amid the COVID-19 Pandemic |
| Mayor | 2022 | Covid-19: Warning over transmission risk as self-isolation is cut to five days in England |
| Meier and others | 2022 | Travel restrictions and variants of concern: global health laws need to reflect evidence |
| Meintrup and others | 2022 | A Comparison of Germany and the United Kingdom Indicates That More SARS-CoV-2 Circulation and Less Restrictions in the Warm Season Might Reduce Overall COVID-19 Burden. |
| Mercer and others | 2022 | The Coronavirus Standards Working Group's roadmap for improved population testing |
| Mezencev and others | 2021 | Stringency of the containment measures in response to COVID-19 inversely correlates with the overall disease occurrence over the epidemic wave |
| Munro and others | 2022 | Face coverings have little utility for young school-aged children |
| Nam and others | 2021 | Early centralized isolation strategy for all confirmed cases of COVID-19 remains a core intervention to disrupt the pandemic spreading significantly |
| Nikolaeva and others | 2022 | Analytical observational study evaluating global pandemic preparedness and the effectiveness of early COVID-19 responses in Ethiopia, Nigeria, Singapore, South Korea, Sweden, Taiwan, UK and USA |
| Olumoyin and others | 2021 | Data-Driven Deep-Learning Algorithm for Asymptomatic COVID-19 Model with Varying Mitigation Measures and Transmission Rate |
| Page and others | 2022 | Computational Simulation Is a Vital Resource for Navigating the COVID-19 Pandemic |
| Pan and others | 2021 | The new UK SARS-CoV-2 variant and lockdown - causes and consequences |
| Peeling and others | 2021 | Rolling out COVID-19 antigen rapid diagnostic tests: the time is now |
| Pei and others | 2022 | Adaptive Multi-Factor Quantitative Analysis and Prediction Models: Vaccination, Virus Mutation and Social Isolation on COVID-19 |
| Peto | 2021 | Weekly population testing could stop this pandemic and prevent the next |
| Piovani and others | 2021 | Effect of early application of social distancing interventions on COVID-19 mortality over the first pandemic wave: An analysis of longitudinal data from 37 countries |
| Prestige and others | 2022 | Covid lockdowns in the UK: Estimating their effects on transmission |
| Pugh and others | 2022 | Sense and sensitivity: can an inaccurate test be better than no test at all? |
| Puspita and others | 2021 | Effectiveness of lockdown in reducing the spread of covid-19 |
| Rehms and others | 2022 | A Bayesian hierarchical approach to account for reporting uncertainty, variants of concern and vaccination coverage when estimating the effects of non-pharmaceutical interventions on the spread of infectious diseases |
| Robertson | 2021 | Did people's behavior after receiving negative COVID-19 tests contribute to the spread? |
| Russell and others | 2021 | Effect of internationally imported cases on internal spread of COVID-19: a mathematical modelling study |
| Sameni | 2021 | Model-based Prediction and Optimal Control of Pandemics by Non-pharmaceutical Interventions |
| Sarma and others | 2021 | Country-specific Optimization Strategy for Testing Through Contact Tracing Can Help Maintain a Low Reproduction Number (R_0) During Unlock |
| Savage and others | 2021 | Social Intervention by the Numbers: Evidence behind the Specific Public Health Guidelines in the COVID-19 Pandemic |
| Sharma and others | 2021 | Understanding the effectiveness of government interventions in Europe’s second wave of COVID-19 |
| Shiraef and others | 2022 | Did border closures slow SARS-CoV-2? |
| Sleat and others | 2021 | Are vaccine passports and covid passes a valid alternative to lockdown? |
| Soljak and others | 2022 | Reducing the covid-19 isolation period in England: a policy change that needs careful evaluation |
| Sopory and others | 2022 | Quarantine acceptance and adherence: qualitative evidence synthesis and conceptual framework |
| Stokel-Walker | 2021 | Covid-19: Why test and trace will fail without support for self-isolation |
| Stokes and others | 2022 | The relative effects of non-pharmaceutical interventions on wave one Covid-19 mortality: natural experiment in 130 countries |
| Sun and others | 2022 | Quantifying the Effect of Public Activity Intervention Policies on COVID-19 Pandemic Containment Using Epidemiologic Data From 145 Countries |
| Tanner and others | 2021 | Increase in circulation of non-SARS-CoV-2 respiratory viruses following easing of social distancing is associated with increasing hospital attendance |
| Thu and others | 2020 | Effect of the social distancing measures on the spread of COVID-19 in 10 highly infected countries |
| Voko and others | 2020 | The effect of social distance measures on COVID-19 epidemics in Europe: an interrupted time series analysis |
| Walker and others | 2021 | Airborne transmission of covid-19: Reduce the viral load in inhaled air |
| Wells and others | 2022 | Quarantine and testing strategies to ameliorate transmission due to travel during the COVID-19 pandemic: a modelling study |
| Wen and others | 2022 | Non-pharmacological interventions of travel restrictions and cancelation of public events had a major reductive mortality affect during pre-vaccination coronavirus disease 2019 period. |
| Wibbens and others | 2021 | Which COVID policies are most effective? A Bayesian analysis of COVID-19 by jurisdiction |
| Wilasang and others | 2021 | Reduction in effective reproduction number of COVID-19 is higher in countries employing active case detection with prompt isolation |
| Wilson | 2020 | Face coverings now required |
| Wood and others | 2021 | Was R < 1 before the English lockdowns? On modelling mechanistic detail, causality and inference about Covid-19 |
| Yu and others | 2021 | What Matters among Non-pharmaceutical Interventions on COVID-19 in Europe? |
| Zhang and others | 2022 | Rethinking Lockdown Policies in the Pre-Vaccine Era of COVID-19: A Configurational Perspective |
| Zhu and others | 2021 | Evolution of disease transmission rate during the course of SARS-COV-2: Patterns and determinants |
| **Wrong publication type n=13** | | |
| Berger and others | 2021 | Phase 2 of the Norwich COVID-19 testing initiative: an evaluation |
| Brophy | 2022 | SARS-CoV-2 testing in travellers: Can we be smarter? |
| Department for Digital, Culture, Media and Sport | 2021 | Events Research Programme. Phase I findings |
| Ferguson and others | 2020 | Impact of non-pharmaceutical interventions (NPIs) to reduce COVID-19 mortality and healthcare demand |
| Fu and others | 2021 | Mathematical Modelling of Lockdown Policy for COVID-19 |
| Godlee | 2021 | Caution, vaccines, testing: The only way forward |
| Guest and others | 2022 | COVID-19 Detection Using the NHS Lateral Flow Test Kit |
| Hinch and others | 2020 | Effective configurations of a digital contact tracing app: a report to NHSX |
| Kyle and others (for Public Health Wales) | 2021 | Self-isolation confidence, adherence and challenges: behavioural insights from contacts of cases of COVID-19 starting and completing self-isolation in Wales |
| McNally | 2022 | Covid-19 testing in the UK was not a “shambles” in 2020 |
| Rutherford and others (for the Scottish Government) | 2021 | COVID-19 support study: experiences and compliance with self-isolation |
| Scientific Pandemic Influenza Group on Behaviours (SPI-B) for the UK government | 2020 | The impact of financial and other targeted support on rates of self-isolation or quarantine |
| University of Liverpool | 2021 | Covid-SMART asymptomatic testing pilot in Liverpool City region: quantitative evaluation |
| **Duplicate reference n=15, references not listed here** | | |
